# Deciphering UBE4B phosphorylation dynamics: a key mechanism in p53 accumulation and cancer cell response to DNA damage

**DOI:** 10.1038/s41420-025-02441-9

**Published:** 2025-04-02

**Authors:** Yasser Abuetabh, H. Helena Wu, Habib Al Yousef, Sujata Persad, Mary-Pat Schlosser, David D. Eisenstat, Consolato M. Sergi, Roger Leng

**Affiliations:** 1 370 Heritage Medical Research Center, Department of Laboratory Medicine and Pathology, University of Alberta, Edmonton, AB T6G 2S2 Canada; 2 Department of Pediatrics, University of Alberta, 11405 - 87 Ave., Edmonton, AB T6G 1C9 Canada; 3 Department of Oncology, Cross Cancer Institute, 11560 University Ave., University of Alberta, Edmonton, AB T6G 1Z2 Canada; 4 Department of Medical Genetics, University of Alberta, 8613 114 Street, Edmonton, AB T6G 2H7 Canada; 5 Murdoch Children’s Research Institute, Department of Paediatrics, University of Melbourne, 50 Flemington Road, Parkville, VIC 3052 Australia; 6 Division of Anatomical Pathology, Children’s Hospital of Eastern Ontario (CHEO), University of Ottawa, 401 Smyth Road Ottawa, Ottawa, ON K1H 8L1 Canada

**Keywords:** Cancer models, Apoptosis

## Abstract

The p53 tumor suppressor protein plays a crucial role in detecting and eliminating various oncogenic threats by promoting processes such as cell cycle arrest, DNA repair, senescence, and apoptosis. UBE4B is essential for negatively regulating p53 during normal conditions and following DNA damage. In previous studies, we demonstrated that UBE4B targets phosphorylated p53 for degradation in response to DNA damage. However, the regulation of UBE4B in relation to DNA damage in cancer is not well understood. In this study, we show that the UBE4B protein is regulated through a phosphorylation and dephosphorylation mechanism in response to DNA damage. Phosphorylation of UBE4B reduces its binding affinity to p53, leading to an accumulation of p53 in the cell. Wip1 plays a crucial role in the dephosphorylation of UBE4B, which stabilizes the activity of the UBE4B protein in response to DNA damage. UBE4B is primarily phosphorylated through ATR-mediated signaling, which reduces its binding affinity with p53, resulting in the accumulation and activation of p53. When Wip1 is inhibited, there is a significant increase in UBE4B phosphorylation, leading to more p53 accumulation and a reduction in cell growth. Therefore, understanding how UBE4B is regulated in cancer cells in response to DNA-damaging agents could help develop new therapeutic strategies to improve the prognosis for cancer patients.

## Introduction

For a century, radiotherapy (a DNA-damaging agent) has been an integral part of the cancer treatment strategy. It is also applied to slow cancer growth and improve patient quality of life with advanced or inoperable cancers [1]. As a result, up to 50% of all cancer patients receive radiation therapy [2]. There are significant variations in the success and failure rates of radiotherapy across different types of cancer with varying genetic profiles. This disparity highlights the need to understand the molecular mechanisms that underpin how this therapy affects cancer cells. Research has revealed many mechanisms and their associated signaling networks that influence the effectiveness of radiation therapy. These discoveries have led to significant advancements in the field. Notably, many of these findings have identified what could be considered the “Achilles’ heel” of targeted cancer cells—specifically, their increased sensitivity to radiation therapy. Consequently, identifying such molecules and their signaling pathways has become an active area of research.

The p53 tumor suppressor is well-known for its role in mediating complex DNA damage response networks [3]. As a transcription factor, p53 activates numerous genes that are essential for various multicellular functions, including cell cycle regulation, DNA repair, cell metabolism, and cell fate [4]. Consequently, p53 is crucial for genome stability and preventing tumor growth. Notably, p53 is one of the most commonly mutated and inactivated tumor suppressor genes found in cancers [5]. During homeostatic and stress conditions, the stability of the p53 protein is regulated primarily by posttranslational modifications (PTM), including phosphorylation, acetylation, and ubiquitination [6].

Our laboratory has been studying the p53 tumor suppressor and its regulatory network in cancers for many years [7–14]. We identified that ubiquitination factor E4B (UBE4B) is essential for HDM2-mediated ubiquitination and degradation of p53 [14]. The regulation of p53 by UBE4B has been well established. Additionally, UBE4B has several substrates, including p73, ataxin-3, ∆Np63α isoform, and EGFR [15–17]. Dysregulation of UBE4B has been observed in various cancers, including those of the brain, liver, breast, and nasopharynx [14, 18, 19]. We further demonstrated that UBE4B binds to and degrades phosphorylated p53 (at S15 and S392) in response to ionizing radiation (IR) [10]. This finding strongly suggests that UBE4B plays a critical role in the regulation of p53 following DNA damage. However, the mechanisms governing UBE4B regulation in response to DNA damage remain unclear.

Wild-type p53-induced phosphatase 1 (Wip1), also known as PPM1D (Protein Phosphatase, Mg2 + /Mn2+ Dependent 1D), plays a crucial role in regulating various molecules in response to DNA damage. This includes p53 and several of its positive and negative regulators [20–27]. Wip1 directly binds to and dephosphorylates both p53 and HDM2. The dephosphorylation of p53 results in its destabilization and eventual degradation. In contrast, dephosphorylation of HDM2 prevents its autoubiquitination and enhances its interaction with unphosphorylated p53. The Wip1-HDM2 axis functions as a negative feedback loop that fine-tunes p53 activity in response to DNA damage. Notably, an important loop between the sustained activation of p53 and transcriptional repression of Wip 1 has been demonstrated [28, 29]. Our laboratory’s research has established two key points regarding this mechanism: (i) HDM2 requires UBE4B to polyubiquitinate p53 under normal physiological conditions [14], and (ii) UBE4B binds directly to phosphorylated p53 and facilitates its proteasomal degradation in response to DNA damage in a manner independent of HDM2 [10]. These findings indicate that UBE4B is a critical molecule that negatively regulates p53 activity and fine-tunes DNA damage responses. Therefore, it is crucial to understand how UBE4B is regulated in the context of DNA damage.

This study shows that the UBE4B protein is regulated through a phosphorylation and dephosphorylation mechanism in response to DNA damage. UBE4B is primarily phosphorylated by upstream ATR-mediated signaling, which reduces its binding affinity for p53 and leads to the accumulation and activation of p53. Additionally, we identified that Wip1 binds to and dephosphorylates UBE4B. The dephosphorylation of UBE4B by Wip1 stabilizes its activity in response to DNA damage. Inhibiting Wip1 results in a significant increase in UBE4B phosphorylation and an accumulation of p53. Furthermore, disrupting the UBE4B-Wip1 axis significantly reduces cell proliferation and growth in response to DNA-damaging agents. Therefore, UBE4B plays a crucial role in the cellular response to DNA damage, and its inhibition may enhance the effectiveness of DNA damage therapies.

## Results

### UBE4B is induced in response to IR and UV treatments

**Fig. 1:**
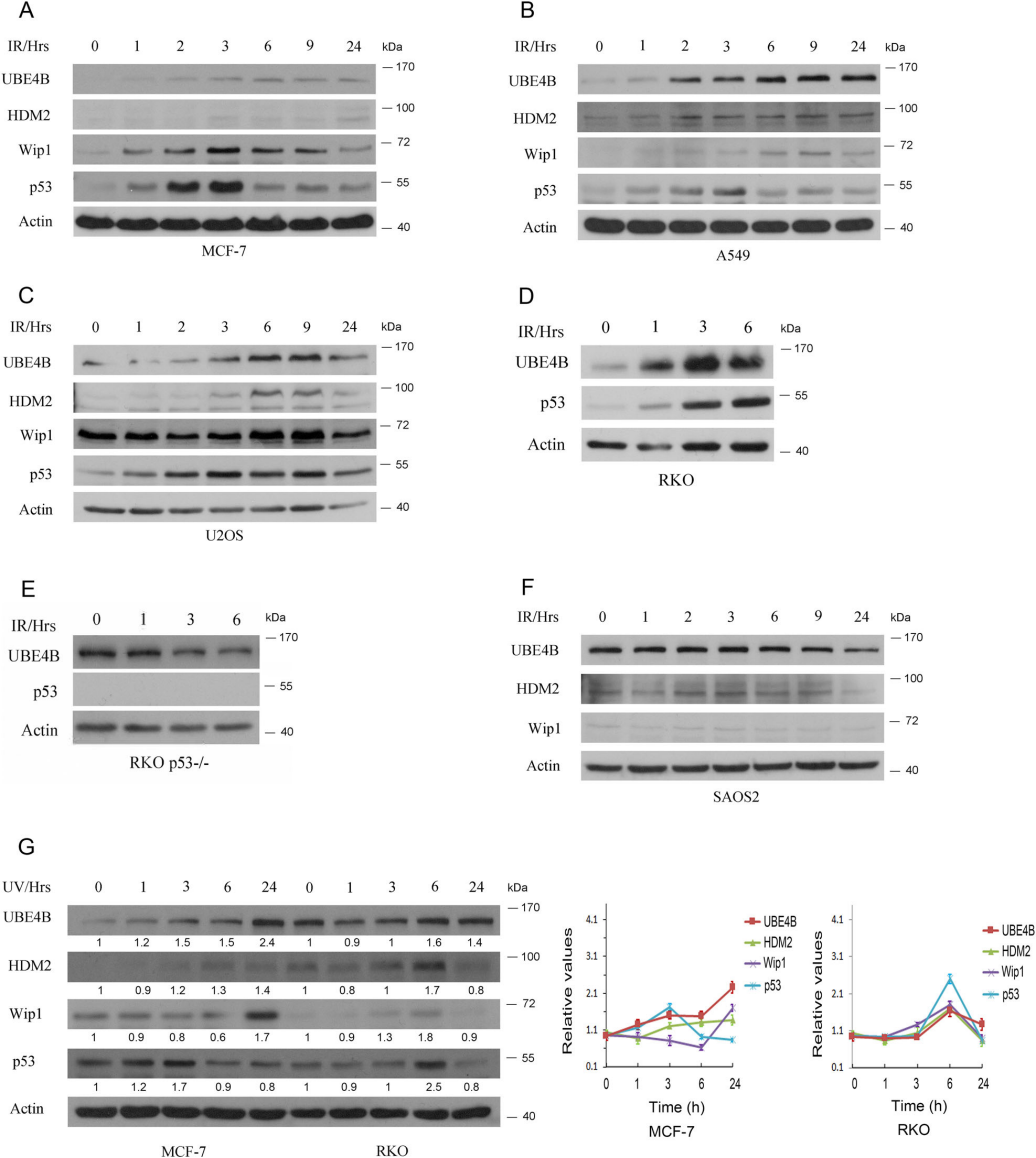
UBE4B is mainly induced in wt-p53 expressing cancer cell lines in response to IR and UV. **A** MCF-7; **B** A549; **C** U2OS; **D** RKO; **E** RKO-p53 ^−/−^; **F** SAOS2. These cells were irradiated with 6 Gy IR and harvested at the indicated time points. UBE4B, HDM2, Wip1, and p53 protein levels were analyzed by immunoblotting. **G** MCF-7 and RKO cells were treated with 10 J/m^2^ UV. These cells were then harvested at the indicated time points. The levels of UBE4B, HDM2, Wip1, and p53 were analyzed by immunoblotting analysis. Actin was used as a loading control. Densitometry was performed using the ImageJ software (NIH), and the relative UBE4B, HDM2, Wip1, and p53 band intensity was normalized to β-actin. An antibody against actin was used as a loading control. Error bars indicate SEM (n-3).

One of the simple techniques to initially identify whether a particular protein may play a role during active cellular and molecular responses to DNA damage is to study variation in protein levels before and after exposure to DNA-damaging agents. Furthermore, it would be highly beneficial to investigate the conservation of this variation between different cell types and in response to varying types of DNA-damaging agents. To investigate the induction of UBE4B in various types of cancer and to gain a better understanding of its role in response to DNA damage caused by ionizing radiation (IR) or ultraviolet (UV), the levels of UBE4B protein were examined in multiple cancer cell lines upon exposure to IR or UV. The dynamic profiles of UBE4B, p53, Wip1, and HDM2 in response to IR in the MCF-7 breast cancer cell line are shown in Fig. 1A. MCF-7 has been widely investigated and used by many laboratories to study the dynamics of different proteins in response to DNA-damaging agents [10, 30–35]. MCF-7 cells harbor the wild-type (wt) p53 protein, which is induced early in response to 6 Gy and reaches the highest level (the peak) at 3 hours after radiation. In addition, the *Wip1* gene was amplified in MCF-7 cells, resulting in increased expression of the Wip1 protein in MCF7 cells [36]. The Wip1 protein peaked at 3 hours after radiation. Furthermore, UBE4B induction in MCF-7 cells is significant, starting at 2 hours after radiation and reaching higher levels at 6 h. In contrast, the levels of MDM2 have not changed significantly in MCF7 cells in response to IR, suggesting that UBE4B may play a more important role in regulating p53 in MCF7 cells in response to IR.

We conducted further studies on the kinetics of UBE4B induction in several human cell lines that express endogenous wild-type p53. This included the cell lines A549 [37], U2OS [38, 39], and RKO [40, 41], all of which were treated with IR. As controls, we also used p53-null cell lines, such as RKO p53^−/−^ colon carcinoma and SAOS2 osteosarcoma cells. Our results showed that UBE4B proteins were significantly induced in response to IR in wild-type p53 cells (Fig. 1B-D), while no induction was observed in p53-null cells after IR treatment (Fig. 1E, F). This indicates that UBE4B induction primarily depends on the presence of p53. Previous studies indicate that p53 mRNA levels are not usually induced after activation in these cell lines [42]. Although UVC radiation is not commonly used clinically in settings like IR, it is still considered a valuable tool for studying the response to DNA damage in laboratory studies [43]. MCF-7 and RKO cells were irradiated with moderate UVC (not a super-lethal dose), and UBE4B levels were evaluated. UBE4B protein levels were elevated in response to UVC treatment in both cell lines (Fig. 1G). The relative quantification of UBE4B, HDM2, Wip1, and p53 over time in MCF-7 and RKO cells were plotted. UBE4B and HDM2 levels gradually increase, while Wip1 decreases slightly, and p53 exhibits moderate fluctuations, suggesting that UBE4B and HDM2 may function together to restrict p53 accumulation under UV-induced stress in MCF-7 cells. In RKO cells, p53 exhibits a sharp peak at 6 hours, followed by a decline at 24 hours. This pattern correlates with an increase in UBE4B and HDM2, suggesting that the induction of UBE4B may contribute to p53 degradation or turnover at later time points. The inverse relationship between UBE4B/HDM2 and p53 at later time points (notably in RKO) supports the hypothesis that UBE4B might act as a p53 regulator through HDM2-mediated ubiquitination and degradation. Wip1 decreases in both cell lines over time, suggesting its potential role in modulating p53 feedback mechanisms under UV-induced stress. Our findings reveal that UBE4B may have different roles in cellular and molecular response programs to DNA damage

### Destabilization of p53 by Wip1 is linked to the upregulation of UBE4B in response to DNA damage

**Fig. 2:**
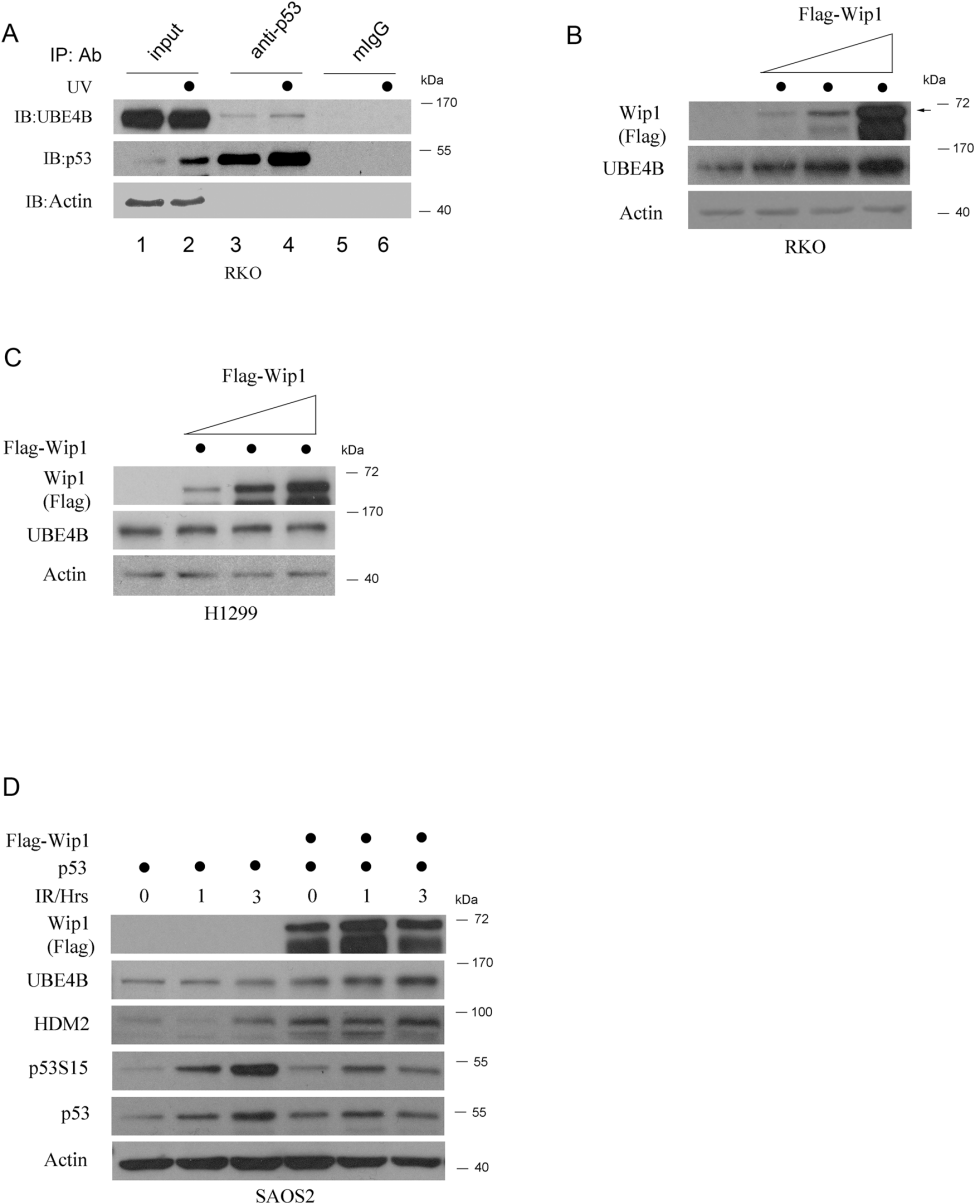
Wip1 expression increases endogenous UBE4B protein. **A** RKO cells were treated with or without 10 J/m2 UV radiation and harvested at 3 hours post-radiation. Cell extracts were immunoprecipitated with p53 (Pab1801) or mouse IgG or p53 antibodies. The levels of UBE4B, p53, and Actin were analyzed by immunoblotting. The dots indicate UV treatment of the samples. For example, lanes 2, 4, and 6 represent UV-treated samples. **B** RKO cells were transiently transfected with 5, 10, and 20 µg of Flag-tagged Wip1-expressing vector. Thirty-six hours post-transfection, total proteins were extracted and analyzed by immunoblotting with antibodies against Flag (Wip1), UBE4B, and Actin. **C** H1299 cells were transiently transfected with 5, 10, and 20 µg of Flag-tagged Wip1-expressing vector. Thirty-six hours post-transfection, total proteins were extracted and analyzed by immunoblotting with antibodies against Flag (Wip1), UBE4B, and Actin. **D** SAOS2 cells were transiently cotransfected with Flag-tagged Wip1 and p53-expressing plasmids. After transfection, cells were treated with or without 6 Gy IR and harvested at 1 and 3 hours. The levels of UBE4B, HDM2, Flag (Wip1), phospho-p53S15, and p53 were detected by immunoblotting. Actin was used as a loading control.

In a previous study, we demonstrated that UBE4B binds to and facilitates the degradation of p53 and its phosphorylated forms at S15 and S392 in response to IR radiation [10]. To further investigate the role of UBE4B in regulating p53, we conducted a co-immunoprecipitation assay using RKO cells to determine whether UBE4B exhibits a similar regulation to p53 in response to UV radiation. Total protein lysates were extracted from RKO cells treated with UV radiation at various time points and immunoprecipitated with a p53 antibody. Our findings indicate that UV radiation enhances the binding affinity between UBE4B and p53 (Fig. 2A). These results demonstrate that UBE4B consistently regulates p53 in response to both IR and UV radiation.

Wip1 is a phosphatase that plays a crucial role in regulating various networks involved in responding to DNA damage. It is possible that Wip1 positively influences UBE4B activity in response to DNA damage. To investigate this hypothesis, we first overexpressed Wip1 and assessed the total endogenous levels of UBE4B following DNA damage. RKO cells were transfected with increasing amounts of Flag-tagged Wip1, and the endogenous levels of UBE4B protein were evaluated through immunoblotting. The results indicated a direct positive relationship between UBE4B and Wip1 (Fig. 2B). However, this relationship appears to depend on p53, as Wip1 overexpression did not influence UBE4B levels in H1299 cells [non-small cell lung cancer (NSCLC) cells], which lack wt-p53 expression (Fig. 2C). Wip1 inhibits the accumulation of p53 and promotes its ubiquitination [24]. To determine further whether Wip1 decreases p53 vis upregulation of UBE4B following DNA damage, SAOS2 cells (lacking p53) were cotransfected with p53 and Flag-tagged Wip1 and irradiated with IR as indicated. As shown in Fig. 2D, Wip1 overexpression significantly increased UBE4B levels, while both total and phospho-p53S15 levels decreased. Furthermore, we noted an increase in HDM2 levels. Together, these findings indicate that Wip1 regulates p53 through UBE4B in response to DNA damage.

### Wip1 physically interacts with UBE4B

**Fig. 3:**
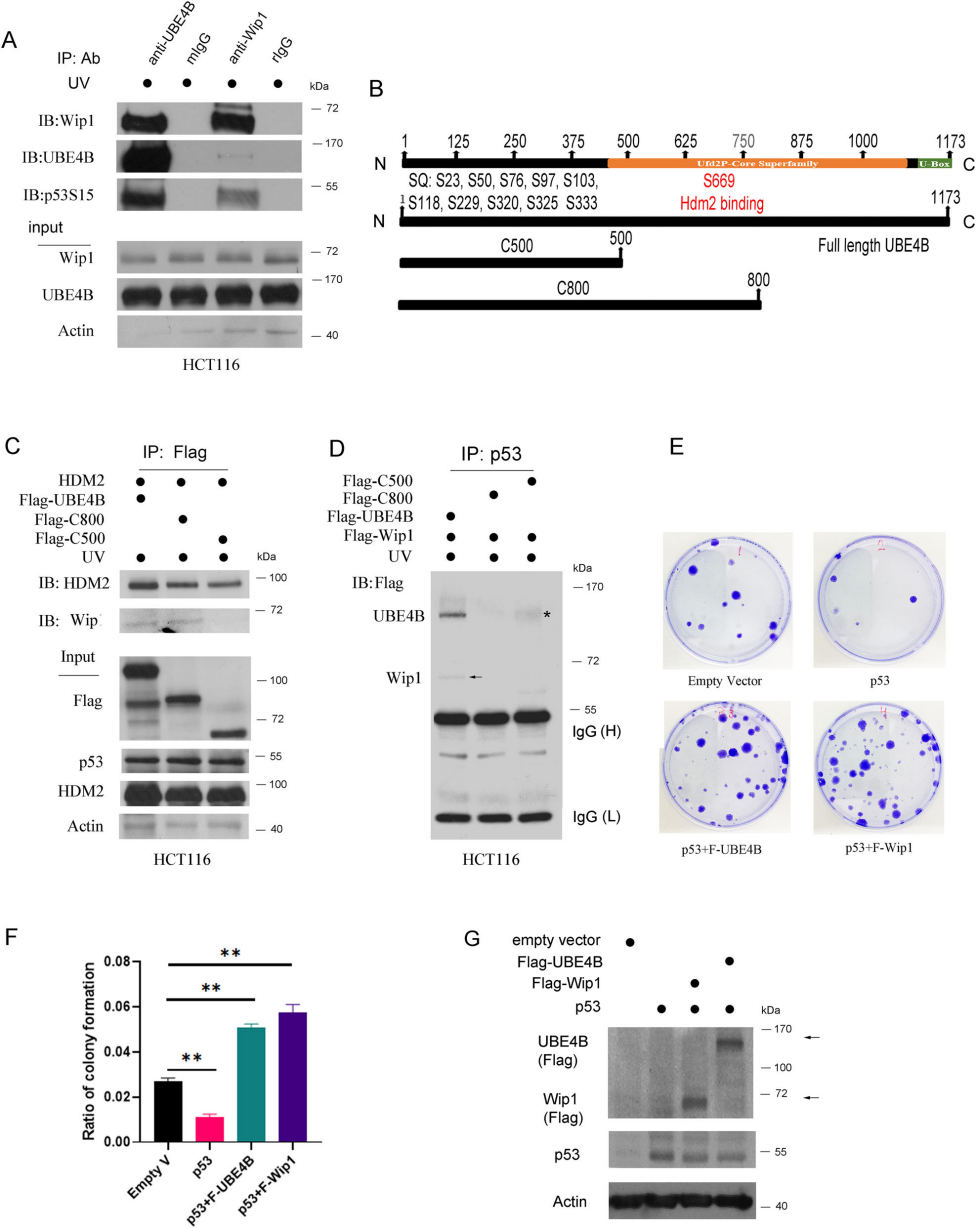
Interaction between Wip1 and UBE4B. **A** HCT116 cells were irradiated with or without 10 J/m^2^ UV and harvested 3 hours post-radiation. Cell extracts (600 to 800 µg of total proteins) were immunoprecipitated with antibodies against UBE4B, Wip1 or IgG [mouse IgG (mIGg) for UBE4B, and rabbit IgG (rIgG) for Wip1]. The levels of UBE4B, Wip1, p53, and phospho-p53S15 were detected by immunoblotting. Approximately 30 to 50 µg of the total proteins used for the immunoprecipitation assay were used as input (direct immunoblotting). **B** We have created three constructs that express UBE4B and UBE4B mutants. 1- Full-length UBE4B construct. 2- C500 construct that contains only the first 500 amino acids of the N-terminal UBE4B. 3- C800 construct that contains the first 800 amino acids of the N-terminal UBE4B. All constructs are Flag-tagged. **C** HCT116 cells were transfected with plasmids expressing HDM2, UBE4B, C500 and C800 as indicated. After transfection, cells were irradiated with 10 J/m^2^ UV and harvested 3 hours after radiation. Cell extracts were immunoprecipitated with an anti-Flag antibody (M5), and the Wip1 protein was detected by immunoblotting. **D** HCT116 cells were transfected with plasmids expressing UBE4B, Wip1, C500, and C800 plasmids. Cells were harvested 3 hours after UV treatment and the cell extracts were immunoprecipitated with an anti-p53 antibody (Pab1801). The UBE4B and Wip1 protein bands were detected by immunoblotting with an antibody against Flag. The asterisk (*) denotes the background in Western blots. **E** SAOS2 cells were transfected with the indicated plasmids. Forty-eight hours later, cells were placed under drug selection (G418) for two weeks. Colonies were fixed with 100% methanol and stained with 0.5% crystal violet. **F** The ratio of colony formation is presented in the graph. The experiments were carried out in triplicate. **P < 0.01. **G** Protein levels of UBE4B (Flag) Wip1 (Flag), and p53 expression were detected by Western blotting with Flag-specific (M5) and p53-specific (Pab1801) antibodies. An antibody against actin was used as a loading control.

Wip1 directly regulates UBE4B’s phosphorylation status and activity in response to DNA damage. Understanding the specific binding interaction between Wip1 and UBE4B is crucial for elucidating the mechanistic basis of this regulation. By mapping the binding site, we aimed to identify the domains or motifs involved in their interaction, which may provide insights into the functional consequences of this interaction on p53 regulation. We conducted a co-IP assay. HCT116 colon carcinoma cells were exposed to 10 J/m² UV radiation. Following this exposure, total cell extracts were co-immunoprecipitated using antibodies specific to UBE4B, Wip1, or IgG (from either mouse or rabbit). The samples were then analyzed by immunoblotting with the indicated antibodies. Consistent with previous findings, we observed that UBE4B was co-immunoprecipitated with Wip1. Moreover, both UBE4B and Wip1 were co-immunoprecipitated with phospho-p53 S15 in response to DNA damage (Fig. 3A). Currently, no commercially available antibodies target phosphorylated UBE4B at any residue. Through in silico analysis, we identified that the UBE4B protein contains 13 potential ATM/ATR phosphorylation sites with SQ motifs, which include S23, S50, S76, S97, and additional residues labeled as S103, S118, S229, S320, S325, S333, S497, S669, and S945. Notably, the serine residue at position 669 is situated in the most conserved region of the core domain found in the UFD2 ligase (Fig. 3B). Two Mdm2-binding domains (amino acids 1–282 and 501–800) of Ube4b were previously mapped [14]. To investigate the region of UBE4B that interacts with Wip1, we generated two truncated UBE4B constructs, C500 and C800 (Fig. 3B). The C500 construct consists of the first 500 N-terminal amino acids of UBE4B, while the C800 construct includes the first 800 N-terminal amino acids. We hypothesized that the C500 construct would not co-immunoprecipitate with Wip1. To test this hypothesis, HCT116 cells were cotransfected with plasmids expressing Flag-tagged UBE4B, HDM2, Flag-tagged C500, and Flag-tagged C800. Thirty-six hours after transfection, the cells were irradiated with UV light and then harvested three hours later. Cell extracts were co-immunoprecipitated using the Flag antibody (M5) and analyzed by immunoblotting. As expected, the C500 construct did not co-immunoprecipitate with Wip1. Only the full-length UBE4B and the C800 construct co-immunoprecipitated with Wip1 (Fig. 3C). In line with our previous report [14], HDM2 is able to bind to all three constructs of UBE4B. This is attributed to the fact that each truncated construct includes amino acids 1–282 of UBE4B. Additionally, HCT116 cells were cotransfected with plasmids that express Flag-Wip1, either alone or in combination with Flag-UBE4B, Flag-C500, or Flag-C800, as indicated. Notably, only the full-length UBE4B was able to facilitate the interaction between p53 and Wip1 in response to UV exposure (Fig. 3D).

SAOS2 cells were transfected with plasmids that expressed either an empty vector, p53, or combinations of p53 with UBE4B or p53 with Wip1. After selection with G418 for two weeks, a colony formation assay was conducted. The results showed that the overexpression of UBE4B or Wip1 significantly increased the colony formation rate, suggesting that both UBE4B and Wip1 promote the proliferation of cancer cells (Fig. 3E, F). The increase in cells that overexpress UBE4B and WIP1, compared to the parental SAOS cells, does not rule out the possibility that these proteins may function through pathways other than p53. UBE4B, WIP1, and p53 protein levels were examined using Western blotting (Fig. 3G).

### Wip1 inhibits the self-ubiquitination of UBE4B and augments UBE4B stability

**Fig. 4:**
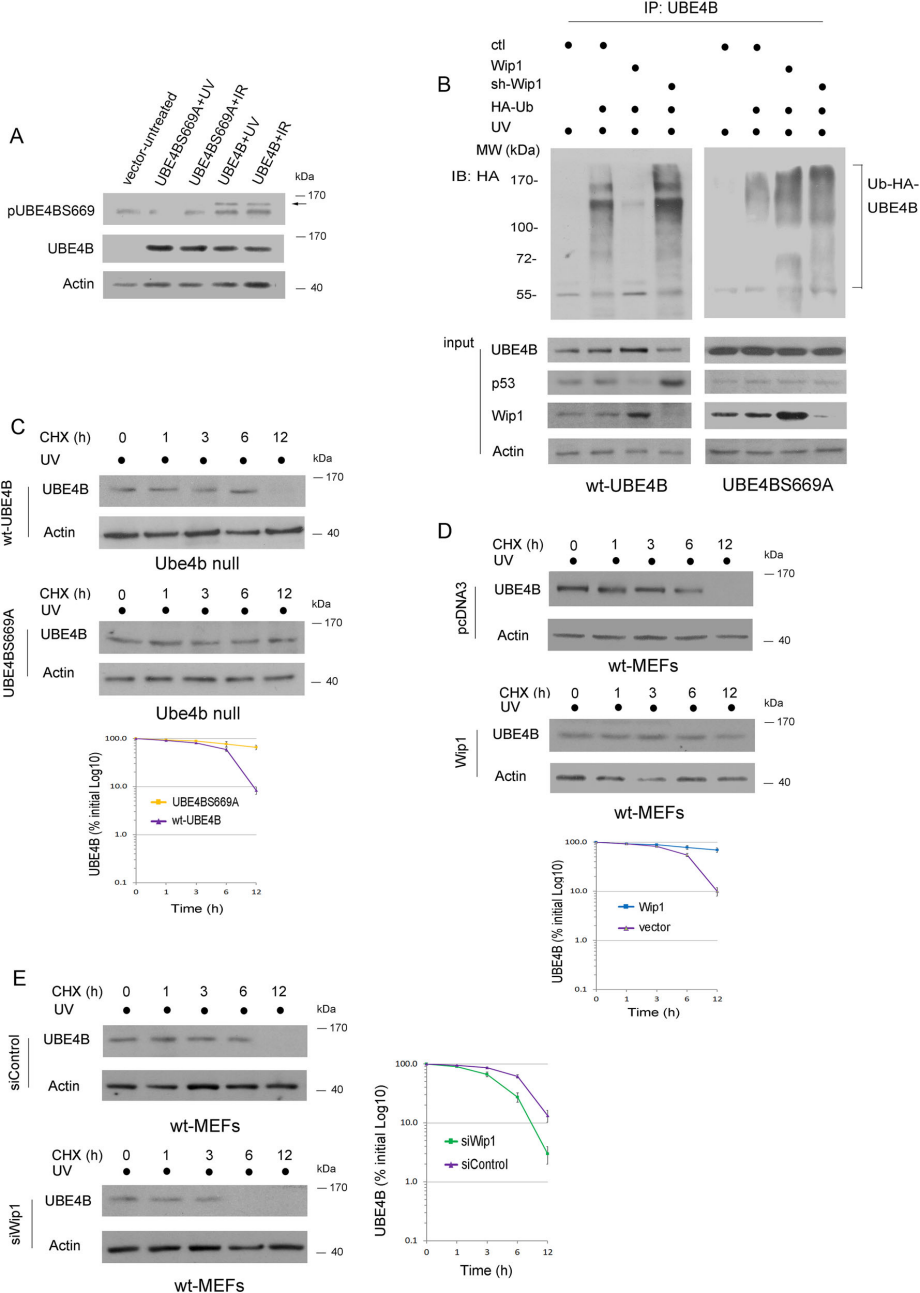
Wip1 inhibits the self-ubiquitination of UBE4B and stabilizes UBE4B. **A** Plasmids expressing empty vector, UBE4BS669A, UBE4B, were transfected into Ube4b-null MEFs as indicated. Transfected cells were treated with 10 J/m^2^ UV or IR (6 Gy). The expression of the UBE4BS669 and UBE4B protein levels was detected by Western blotting as indicated. Actin was used as a loading control. **B** Ube4b-null MEF clones stably expressing wt-UBE4B or UBE4BS669A were transfected with plasmids expressing HA-ubiquitin (Ub), Flag-Wip1, Wip1-shRNA as indicated. The transfected cells were then treated with 10 J/m^2^ UV. Cell extracts were immunoprecipitated with a UBE4B-specific antibody and analyzed by immunoblotting with antibodies against HA (12CA5). Direct Western blots for UBE4B, p53 (Pab421), and Wip1 are shown in the lower panels, as indicated. Actin was used as a loading control. **C** Ube4b null MEF clones stably expressing UBE4B or UBE4BS669A were treated with cycloheximide (CHX, 20 µg/ml) and followed by UV treatment (10 J/m^2^). UBE4B protein levels were detected by immunoblotting. Densitometry was performed using the ImageJ software (NIH), and the relative UBE4B band intensity was normalized to β-actin. An antibody against actin was used as a loading control. Error bars indicate SEM (n-3). **D** wt-MEFs were transfected with an empty vector (pcDNA3) or Wip1 expression vector in the presence of CHX and followed by UV treatment. UBE4B protein levels were determined by immunoblotting. Densitometry was performed. Error bars indicate SEM (n-3). **E** wt-MEFs were transfected with either control-shRNA or Wip1-shRNA (siWip1) in the presence of CHX and followed by UV treatment. The UBE4B protein levels were then detected using immunoblotting. Densitometry was performed, and the relative UBE4B band intensity was normalized to β-actin. The error bars indicate the standard error of the mean (SEM), and the experiment was performed in triplicate (*n* = 3). For **C,** **D**, and **E**, the ‘0 h’ timepoint represents samples collected immediately before CHX addition (as non-treated control).

The residue denoted as S669 in UBE4B is of primary interest because it is situated in the most conserved region of the core of the UFD2P ubiquitin ligase. Additionally, this residue is located within one of the binding areas [14]. In particular, UBE4B protein levels were significantly upregulated in response to IR and UV in several human cancer cells carrying wild-type p53 expression (Fig. 1). The phospho-specific antibody for UBE4BS669 was generated and tested by GeneScript (USA). UBE4BS669A (Ser669 is substituted by alanine) was generated by site-directed mutagenesis (Agilent). S669A is considered inactive [44]. Plasmids expressing UBE4BS669A, UBE4B, or empty vector were transfected into Ube4b-null MEFs [14]. The transfected cells were then treated with 10 J/m^2^ UV or 6 Gy IR and analyzed by Western blotting. As shown in Fig. 4A, UBE4B phosphorylation at serine 669 is detected by a pUBE4BS669 phospho-specific antibody in cells transfected with wild-type UBE4B following UV and IR treatment. In contrast, the UBE4BS669A mutant, which lacks this phosphorylation site, does not react with the antibody, indicating the specificity of the antibody for the phosphorylated form of serine 669. This specificity is further confirmed by the absence of antibody reactivity in cells transfected with an empty vector after DNA damage, underscoring the precise detection of phosphorylated UBE4B only. Notably, UBE4BS669A remains more stable (higher protein levels) than wild-type UBE4B after DNA damage, which suggests that phosphorylation at S669 is important for regulating UBE4B stability in response to DNA damage.

We then investigated whether Wip1 affects the self-ubiquitination of UBE4B. We generated Ube4b null MEFs that stably expressed wild-type UBE4B or UBE4BS669A mutant. Plasmids expressing HA-tagged ubiquitin, Wip1, and Wip1-shRNA (sh-Wip1) or control-shRNA were transfected into the Ube4b null stable clones as indicated. The transfected cells were then treated with UV (10 J/m^2^), immunoprecipitated with a UBE4B-specific antibody, and analyzed by Western blotting with an anti-HA antibody to detect ubiquitinated UBE4B4B or with a UBE4B-specific antibody to detect total UBE4B (Fig. 4B). Overexpression of Wip1 reduced the level of self-ubiquitination of UBE4B, while depletion of Wip1 increased it, compared to the control group in wt-UBE4B stable clone (Fig. 4B, top, left panel). In contrast, UBE4BS669A ubiquitination was not significantly affected by Wip1 overexpression or depletion, suggesting that S669 is one of the major phosphorylation sites regulated by Wip1 (Fig. 4B, top, right panel). Notably, the decrease in UBE4B in the presence of siWip1 was accompanied by an increase in the level of endogenous p53 protein. Therefore, our findings indicate that Wip1 increases UBE4B stability by inhibiting its self-ubiquitination.

We then evaluated the half-lives of wild-type (wt) and mutant forms of UBE4B in Ube4b null clones that stably expressed wt-UBE4B or UBE4BS669A when subjected to UV treatment (10 J/m^2^). The half-life of wt-UBE4B in Ube4b null clones that expressed wt-UBE4B was approximately 9 h, and in Ube4b null clones that expressed UBE4BS669A, it was extended to 12 h. This indicates that the phosphorylation status of UBE4B (altered by the S669A mutation) affects the protein stability or expression under DNA damage conditions (Fig. 4C). Wip1 overexpression increased the stability of UBE4B compared to UBE4B in the presence of an empty vector when transfected cells were treated with UV (10 J/m^2^) (Fig. 4D). To investigate the role of Wip1 in a more physiological setting; endogenous Wip1 was subjected to ablation by siRNA, and evaluated its effect on endogenous UBE4B steady-state levels. Wip1 depletion decreased the half-life of UBE4B to approximately 3 h, while the half-life of endogenous UBE4B in the presence of the control siRNA was approximately 9 h when the cells were treated with UV (10 J/m^2^) (Fig. 4E). Together, our findings demonstrate that Wip1 regulated the stability of UBE4B protein after DNA damage.

### The UBE4B protein levels elevate in both ATM-proficient and ATM-deficient lymphoblastoid cells upon exposure to UV radiation

**Fig. 5:**
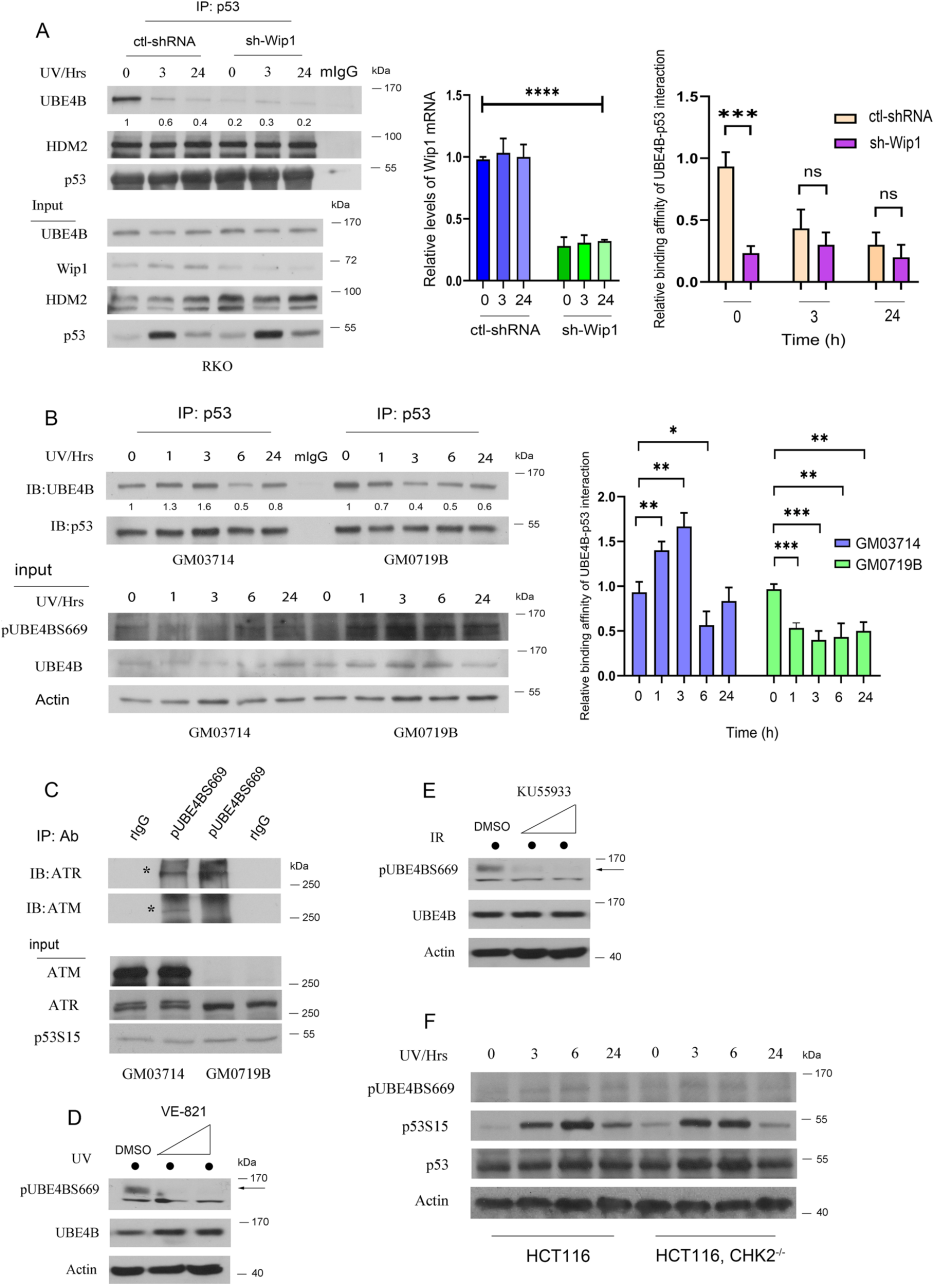
The phosphorylation of UBE4B by ATR facilitates its dissociation from p53 in response to UV exposure. **A** RKO cells that stably expressed a control siRNA (empty vector) or shWip1 were irradiated with UV and harvested at the indicated time points. Cell lysates were immunoprecipitated with an anti-p53 antibody (Pab1801) or mIgG as a control. The levels of UBE4B, Wip1, HDM2, and p53 were detected by immunoblotting. Knockdown efficiency was further determined by qRT-PCR. Error bars indicate SEM (*n* = 3). ****P < 0.0001. Densitometry was performed using the ImageJ software (NIH), and the relative binding affinity between UBE4B-p53 interaction was normalized to IgG and plotted (right panel). The ‘0 h’ timepoint represents samples collected immediately before UV treatment (as non-treated control). **B** GM03714 and GM0719B cells were irradiated with UV light and harvested at the indicated time points. Cell lysates were immunoprecipitated with an anti-p53 antibody (Pab1801) or mIgG as a control. The levels of UBE4B, phosphorylated UBE4B, p53, and phosphorylated p53 were detected by immunoblotting. Densitometry was performed using the ImageJ software (NIH), and the relative binding affinity between UBE4B-p53 interaction was normalized to IgG and plotted (right panel). The ‘0 h’ timepoint represents samples collected immediately before UV treatment (as non-treated control). **C** GM03714 and GM0719B cells were irradiated with or without UV light and harvested 3 hours after radiation. The cell lysates were immunoprecipitated with anti-phosphorylated UBE4B antibody (pS669) or rabbit IgG as a control. The expression of ATM and ATR proteins was detected by immunoblotting. **D** GM0719B cells were preincubated with increased amounts of VE-821 (10–25 µM) or DMSO for 30 min, followed by UV irradiation, and analyzed by immunoblotting. Western blots with indicated antibodies detected the expression of UBE4BS669, UBE4B (total), and actin. **E** Similarly, GM03714 cells were preincubated with increased KU55933 (10–20 µM) or DMSO for 30 min, followed by UV irradiation, and analyzed by immunoblotting. The expression of UBE4BS669, UBE4B (total), and actin was detected by Western blotting, as indicated. **F** Wild-type HCT116 and HCT116 CHK2^−/−^ cells were irradiated with 10 J/m^2^ UV. Cells were harvested at 0, 3, 6, and 24 hours after radiation. The levels of phosphorylated UBE4B and phosphorylated p53 were analyzed by immunoblotting. Actin was used as a loading control.

To determine whether the increase in phosphorylated p53 was due to Wip1 inhibition and DNA damaging agents caused by the UBE4B-p53 disassociation, cell extracts of RKO stable clones expressing sh-Wip1 or a control shRNA vector treated with UV for different times were immunoprecipitated with a p53 specific antibody (Pab1801) to assess the binding affinity between p53 and UBE4B (Fig. 5A). To confirm Wip1 knockdown, we performed qRT-PCR analysis to assess the mRNA levels of Wip1 in RKO cells stably expressing sh-Wip1 or control shRNA. Our data indicates a significant reduction of Wip1 mRNA levels in the sh-Wip1 samples (Fig. 5A, right panel). Relative binding affinity between UBE4B-p53 interaction was plotted.

UBE4B levels in the IP fractions decrease over time in both conditions, but the decrease is more pronounced in sh-Wip1 cells, suggesting that Wip1 depletion reduces UBE4B-p53 interaction. HDM2 and p53 levels appear unchanged across conditions, indicating that the reduced UBE4B signal is not due to changes in p53 input levels. Additionally, UBE4B levels remain relatively stable in the input, indicating that the loss of UBE4B in the IP fraction is due to reduced p53 binding, not lower UBE4B expression. Notably, Wip1 depletion reduces UBE4B-p53 binding under normal conditions (0 h), suggesting that Wip1 is necessary for maintaining UBE4B-p53 interaction. Wip1 may promote UBE4B-mediated ubiquitination of p53, facilitating p53 degradation. After UV exposure, UBE4B-p53 interaction decreases over time in both conditions, indicating that UV stress may disrupt UBE4B’s ability to bind p53, potentially protecting p53 from degradation. Our findings reveal that Wip1 positively regulates UBE4B-p53 interaction. Upon UV exposure, UBE4B dissociates from p53, potentially stabilizing p53. Wip1 knockdown accelerates this dissociation, leading to reduced UBE4B-p53 interaction even before stress. These findings suggest that Wip1 plays a role in controlling p53 stability by modulating its interaction with UBE4B.

The ataxia telangiectasia mutated (ATM) and ataxia telangiectasia and Rad3-related protein (ATR) are critical components of the DNA damage response network that is needed to maintain genomic integrity. Double-stranded DNA breaks mainly trigger ATM activation, while ATR responds to a broader range of DNA damage (ssDNA, such as UV-induced DNA damage). To determine whether ATM does not strictly regulate UBE4B induction in response to IR treatment, cell extracts of GM03714 (B lymphoblasts, proficient in ATM and transformed with EBV) and GM0719B (deficient in ATM) were irradiated with UV, harvested at different times, and immunoprecipitated with p53-specific antibodies. Interestingly, the binding affinity between p53 and UBE4B increased three hours after radiation in GM03714, whereas in GM0719B cells, it drastically reduced (Fig. 5B). Relative binding affinity between UBE4B-p53 interaction was quantified and plotted (Fig. 5B, right panel).

Furthermore, the cell extracts were subjected to direct immunoblotting, which revealed a significant elevation in phosphorylated UBE4B levels following UV exposure in GM0719B cells. This suggested that UBE4B phosphorylation decreases its binding affinity to p53 in response to UV treatment. Our findings indicate that ATR predominantly mediates UBE4B. A co-IP assay was performed to investigate whether phosphorylated UBE4B binds to ATM and ATR. Cell extracts from GM03714 and GM0719B were irradiated with UV, harvested at 3 hours after radiation, and immunoprecipitated with the anti-phospho-UBE4BS669 antibody. Immunoblotting revealed that phospho-UBE4B was co-immunoprecipitated with ATM and ATR, and the latter had a higher affinity (Fig. 5C).

VE-821 is a potent ATR inhibitor that has been shown to effectively suppress a variety of solid malignancies [45–49]. It has been found to induce apoptosis, chromosome fragmentation, and cell death in vitro and exhibit strong anti-cancer activity. We then investigated whether the inhibitory effect of VE-821 on ATR affected the phosphorylation of UBE4BS669 after UV treatment. GM0719B cells were pretreated with VE-821 (10–25 µM) or DMSO for 30 min, followed by UV irradiation. Cell lysates were analyzed by immunoblotting. As shown in Fig. 5D, UBE4B was phosphorylated and detected by pUBE4BS669 phospho-specific antibody after UV treatment. Phosphorylation of UBE4BS669 was effectively inhibited in GM0719B cells treated with VE-821. KU55933 is an ATM-specific inhibitor [50, 51]. Treatment of GM03714 cells with KU55933, which blocks the ATM kinase pathway, largely inhibited the phosphorylation of UBE4BS669 (Fig. 5E). Taken together, our findings demonstrated that ATM and ATR can mediate the phosphorylation of UBE4B at S669. The absence of ATM in GM0719B cells suggests that the ATM-CHK2 pathways may not be necessary for the phosphorylation of UBE4B. To explore whether CHK2 loss affects UBE4B phosphorylation, we used an isogenic pair of HCT116 cell lines [HCT116-WT, and HCT116, CHK2 Knockout, which was purchased from Horizon Discovery (HD R02-017, Waterbeach, Cambridge, UK)]. These cells were irradiated with UV, harvested at different time points, and analyzed by immunoblotting with antibodies against phospho-UBE4B S669, phospho-p53 S15, and total p53. Our results indicate that the loss of CHK2 has no effect on phosphorylated UBE4B levels (Fig. 5F).

### Depletion of UBE4B and Wip1 inhibits cancer cell proliferation

**Fig. 6:**
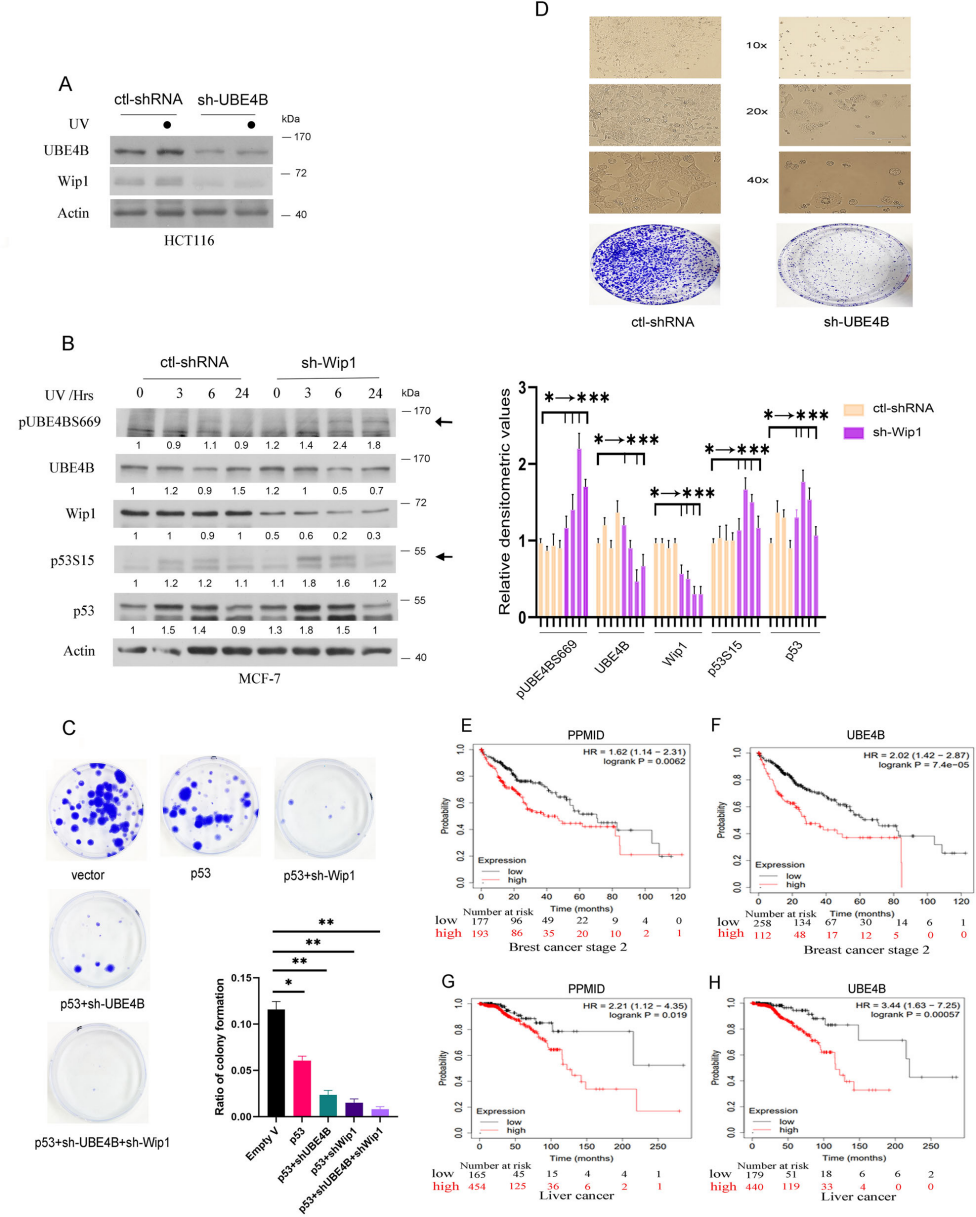
Ablation of UBE4B or Wip1 by shRNA suppresses cell proliferation. **A** HCT116 cells were transfected with empty vector or shUBE4B expression plasmids. Thirty-six hours after transfection, cells were treated with or without UV and harvested 3 hours later. The levels of UBE4B and Wip1 were analyzed by immunoblotting. Actin was used as a loading control. The dots indicate UV treatment of the samples. **B** MCF-7 clones stably expressing control siRNA (empty vector) or shWip1 were irradiated with UV and harvested at the indicated time points. The levels of phosphorylated UBE4B, UBE4B (total), Wip1, p53, and phosphorylated p53 (p53S15) were detected by immunoblotting analysis. Densitometry was performed using the ImageJ software (NIH), and the relative pUBE4BS669, UBE4B, Wip1, p53S15, and p53 band intensity was normalized to β-actin (right panel). The ‘0 h’ timepoint represents samples collected immediately before UV treatment (as non-treated control). **C** H1299 cells were transfected with the indicated plasmids. Forty-eight hours later, cells were treated with G418 for two weeks. Colonies were fixed with 100% methanol and stained with 0.5% crystal violet. The ratio of colony formation is presented in the graph. The experiments were performed in triplicate. *P < 0.05, **P < 0.01. **D** HCT116 clones stably expressing a control-siRNA (empty vector) or shUBE4B were subjected to UV. Cells were incubated for one week and analyzed under a microscope with three levels of magnification: 10x, 20x, and 40x. **E–H** The Kaplan-Meier survival curves revealed that patients with breast cancer (**G** and **H**) and liver cancer (**I** and **J**) with a high level of Wip1 (PPMID) and UBE4B had a significantly lower overall survival time (www.kmplot.com).

Next, we wanted to investigate whether the depletion of UBE4B by siRNA can affect the expression of Wip1. HCT116 cells were transfected with a control-shRNA vector or UBE4B-shRNA (sh-UBE4B). Thirty-six hours after transfection, cells were irradiated with 10 J/m^2^ UV and harvested three hours later. Interestingly, the ablation of UBE4B led to the down-regulation of Wip1 (Fig. 6A).

To study the effect of Wip1 knockdown on phosphorylated UBE4B in cancer cell line, MCF-7 stable clones expressing Wip1-shRNA (sh-Wip1) or ctl-shRNA were exposed to 10 J/m^2^ UV and harvested at different time points, and analyzed by immunoblotting with antibodies against phospho-UBE4B S669, UBE4B, Wip1, p53, and phospho-p53 S15. Our findings revealed that phospho-UBE4B is significantly increased in response to UV treatment when the cells were treated with sh-Wip1 compared to that of ctl-shRNA (Fig. 6B). The relative band intensities of pUBE4BS669, UBE4B, Wip1, p53S15, and p53 were quantified and plotted (Fig. 6B, right panel). Phosphorylation of UBE4B is accompanied by a significant increase in both total and phosphorylated p53. Consistently, shRNA ablation of UBE4B or Wip1 greatly decreased the colony formation ratio (Fig. 6C). Cell proliferation was significantly inhibited by the depletion of both UBE4B and Wip1. Furthermore, HCT116 stable clones expressing a control shRNA vector or UBE4B shRNA were subjected to UV treatment, and analyzed under a microscope. Cell growth treated with UBE4B-shRNA was significantly suppressed compared to cells transfected with a control-shRNA vector (Fig. 6D). Together, our findings indicate that the depletion of UBE4B or Wip1 suppresses cancer cell proliferation.

**Fig. 7:**
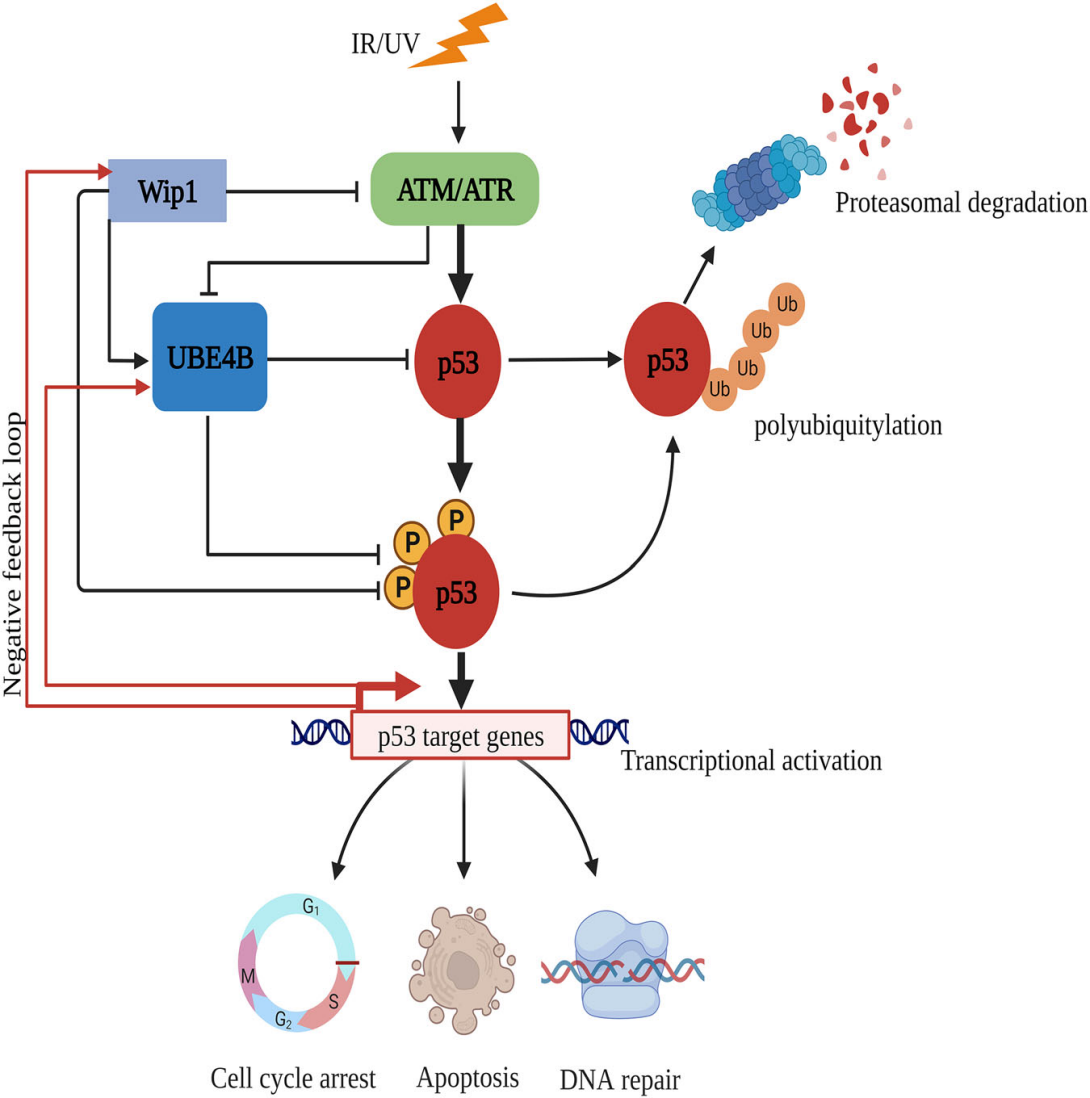
Proposed model of UBE4B regulation in response to DNA damage. UBE4B mediates HDM2-dependent polyubiquitination of p53 to keep its level low during homeostasis. In response to DNA damage, UBE4B activity is prevented by its phosphorylation mediated by ATR/ATM kinases, which decreases the affinity binding of UBE4B-p53 and leads to the accumulation and activation of p53. However, Wip1 dephosphorylates, stabilizes, and activates UBE4B in response to DNA damage. UBE4B binds to and degrades phosphorylated p53. This figure was created using BioRender.com (granted a license “Academic License Terms” no. ET24RT4ROC).

Next, we sought to determine whether there are differences in overall survival between UBE4B and Wip1 expression in human cancers. Kaplan–Meier survival analysis was performed. Kaplan–Meier survival curves revealed that patients with breast cancer and liver cancers with a high level of Wip1 (PPMID) and UBE4B had a significantly lower overall survival time (Fig. 6E–H, kmplot.com [52]). It suggests that the expression of Wip1 and UBE4B in the two human malignancies is likely correlated. We propose a model to elucidate UBE4B regulation in response to DNA damage in the p53 signaling pathway (Fig. 7).

## Discussion

Dynamic behavior (including variation in levels, PTM, cell location, and activities) of a particular protein in response to DNA damaging agents is a valuable technique to initially identify whether a determined protein may play a role during an active cellular and molecular response to DNA damage [53, 54]. The dynamic behavior of p53 and its regulator has been extensively studied. For example, HDM2 protein levels are dramatically reduced early in response to DNA damage. Pulsatile patterns follow this initial decrease. Consequently, the rapid decline in HDM2 is mediated by phosphorylation and autoubiquitination. It allows p53 to dissociate from HDM2 and induce the accumulation of p53 [55]. Here, we investigate the dynamic patterns of UBE4B after DNA damage in several human cancer cell lines. Our data further indicate that UBE4B induction is primarily dependent on p53, as observed in cell lines with wt-p53 but not in p53-null cell lines such as RKO-p53^−/−^ cells and SAOS2 cells. This choice of cell lines allowed us to compare the effects of UBE4B on wt-p53 in diverse genetic backgrounds and to demonstrate its role across different cellular contexts. We concluded that UBE4B plays one or more roles during the active response to DNA damage in a p53-dependent manner.

Under physiological conditions, the p53 protein is continuously degraded by the cooperation of several ubiquitin ligases, including HDM2, Pirh2, UBE4B, and other ubiquitin ligases E3/E4 [14, 42, 56]. Previous studies suggest that phosphorylation of the p53 protein prevents its ubiquitination [57]. However, a study reported that CARPs (caspase 8/10 associated RING proteins) bind to and degrade phosphorylated p53 at S20 [58]. We previously showed that UBE4B binds to and degrades phosphorylated p53 at Serines 15 and 392 in response to IR [10]. In this study, we observed that UBE4B coimmunoprecipitated with p53 in response to UV treatment. The binding affinity of UBE4B-p53 increased consistently after UV treatment, suggesting that UBE4B could play the same regulatory role as phosphorylated p53 in response to IR and UV. UBE4B has been found to coordinate the repair of double-strand break (DSB) and apoptotic pathways in *C. elegans* [59]. Furthermore, UFD2 primarily mediates cell survival in response to cellular stress in yeast [60]. UBE4B was shown to be localized at the DSB site in HCT116 cells [61]. Together, these data indicated that (i) the central role of UBE4B in response to DNA damage is to fine-tune the activity of p53 as a negative feedback loop, and (ii) in addition to p53 regulation, UBE4B can mediate other DNA damage response pathways. Therefore, it is crucial to investigate and understand how UBE4B is regulated in response to DNA damage. Phosphorylation is a critical PTM that governs many protein activities in response to DNA damage. Next, we investigated the PTM status of UBE4B during DNA damage treatment. Phosphorylation of p53 and HDM2 occurs simultaneously and often by the same upstream protein kinases, but leads to opposite results [57]. Many other molecules directly involved in the response to DNA damage undergo phosphorylation, including the E3 ubiquitin ligases COP1, MdmX, and TRIM24 [20, 62–64]. Therefore, phosphorylation is a critical PTM that can modulate UBE4B activity in response to DNA damage. Using bioinformatic strategies, we identified more than a dozen serine residues in UBE4B, which ATM and ATR could potentially phosphorylate. Rabbit polyclonal antibody was generated against S669. The S669 is located in the most conserved part of the core region contained in the UFD2 ligase, and this region is required for binding to HDM2. The roles played by the ATM-CHK2 and ATR-CHK1 pathways in modulating and amplifying DNA damage signals have been well characterized and investigated [20, 62–64]. ATM and ATR primarily target their substrates for phosphorylation in the S/T-Q motif (Ser or Thr followed by Gln) [65]. The residue S669 of UBE4B is followed by glutamine. Phosphorylated UBE4B was co-immunoprecipitated with ATM and ATR in response to DNA damage. Although phosphorylated UBE4B co-immunoprecipitated with ATM and ATR, it appeared that phosphorylated UBE4B bound with ATR with higher affinity. Interestingly, a previous investigation demonstrated that ATM is dispensable in p53 phosphorylation in response to IR. Notably, a higher level of phosphorylated UBE4B is associated with a higher level of p53 in response to DNA damage in ATM-deficient cells (GM0719B). Furthermore, an increased level of phosphorylated UBE4B in ATM-deficient cells is associated with a dramatic decrease in the binding affinity in response to DNA damage. In response to DNA damage, cells with ATM-proficient levels consistently displayed reduced UBE4B phosphorylation and higher UBE4B-p53 binding affinities. UBE4BS669 phosphorylation is inhibited by VE-821 and KU55933 in response to DNA damage. Therefore, a functional ATM alone is insufficient to facilitate UBE4B phosphorylation in response to DNA damage. CHK2 is a primary downstream target of ATM and often has overlapped targets to amplify DNA damage response (DDR) transduction signals [66]. Notably, the loss of the CHK2 gene in HCT116 cells did not affect UBE4B phosphorylation in response to UV. Wip1 deprivation in different cancer cell lines increased in phosphorylated UBE4B at S669 in response to DNA damage (IR and UV). Furthermore, there was a direct correlation between the increase in UBE4B phosphorylation and the increase in the disassociation of UBE4B-p53, resulting in enhanced p53 activity. We confirm that UBE4B co-immunoprecipitated with Wip1. We observe that residue S669 is required for the UBE4B-Wip1 interactions using truncated forms of UBE4B. p53 ubiquitination levels were investigated in response to DNA damage and Wip1 expression, revealing a Wip1-dependent increase in UBE4B-mediated p53 ubiquitination. Wip1 overexpression significantly increased p53 ubiquitination in the presence of DNA damage. These data demonstrated that (i) UBE4B is a substrate for Wip1 and (ii) the UBE4B phosphorylation/dephosphorylation process modulates its activity in response to DNA damage. Notably, we found that inhibition of UBE4B leads to a significant decrease in Wip1 levels.

Interestingly, a published work suggested that UBE4B phosphorylation could inhibit its activity and lead to the arrest of the G2 cell cycle. Phosphorylated UBE4B was detected by analyzing the molecular weight of different UBE4B proteins [67]. The location and activity of Ufd-2 are required at the DNA damage site of germ cells treated with IR in *C. elegans* to release RAD51 from the damage site. The Ufd-2 foci at the damage site modulate the activation of pro-apoptotic pathways after exposure to IR in *C. elegans*. This interaction is crucial for substrate degradation [68]. The Ufd2-RAD23 interaction facilitates substrate degradation. UBE4A, a homolog of UBE4B, was found to form foci after DNA damage, which is necessary to recruit RAP80 and BRCA1 and efficiently repair DSB [69]. Recently, UBE4B foci were detected in response to DNA damage in HCT116 cells [61]. Therefore, it is highly plausible that UBE4B can modulate different pathways that involve DNA repair, cell cycle arrest, and cell fate in response to DNA damage. Specifically, targeting UBE4B and its network can sensitize cancer cells to DNA-damaging agents. We showed that inhibition of UBE4B and Wip1 significantly led to decreased cell proliferation and growth inhibition in response to the DNA-damaging agent UV. Overall survival rates were significantly lower in human liver cancer and breast cancer with UBE4B and Wip1 overexpression.

In this study, we focus on deciphering the mechanistic regulation that governs the stability and activity of UBE4B in response to DNA damage. For the first time, we demonstrate that UBE4B is predominantly phosphorylated through upstream ATR-mediated signaling, which decreases the affinity binding of UBE4B-p53 and leads to the accumulation and activation of p53. In particular, dephosphorylation of UBE4B by Wip1 stabilizes the activity of the UBE4B protein in response to DNA damage. In contrast, depletion of Wip1 led to a significant increase in UBE4B phosphorylation, p53 accumulation, and cell growth inhibition.

## Materials and methods

### Cancer cell lines

HEK293T (RRID: CVCL_0063) cells and several human cancer cell lines have been used and maintained in our laboratory, including MCF-7 (RRID:CVCL_0031), U2OS (RRID: CVCL_0042), A549 (RRID: CVCL_0023), SAOS2 (RRID: CVCL_0548), RKO (RRID: CVCL_0504), RKO, p53^−/−^ (RRID: CVCL_HE25), H1299 (RRID: CVCL_0060), HCT116 (RRID: CVCL_0291), GM03714 (RRID: CVCL_7400), and GM0719B. HEK293T, MCF-7, U2OS, A549, SAOS2, H1299, and HCT116 cells were obtained from the American Type Culture Collection (Manassas, VA, USA). The HCT116, CHK2^−/−^ cell line was ordered from Horizon Discovery Ltd (Cambridge, UK, Cat # HD R02-017). Cell lines RKO and RKO, p53^−/−^ were obtained from the Johns Hopkins School of Medicine (Dr. B. Vogelstein, USA). Cell lines GM03714 and GM0719B were obtained from Dr. R. Mirzayans (Cross Cancer Institute, Edmonton, AB, Canada). All cell lines were grown in Dulbecco’s modified Eagle’s medium (DMEM, Gibco Life Technologies Corp, USA) supplemented with 10% fetal bovine serum (FBS, Sigma Aldrich, USA) and antibiotics. All cell lines were incubated at 5% CO2 and 37 °C in a humidified incubator (Thermo Fisher, USA). All cells were regularly tested using a PCR- based method for mycoplasma. All cell lines were authenticated by short tandem repeat (STR) profiling and PCR-based methods for KO cell lines in the three years prior to experiments. Genomic DNA was prepared from cell lines, primers for the STR sequence were designed, and PCR-amplified. The results of STR typing were compared with the STR database. All cell lines were maintained according to the manufacturer’s source instructions.

### Ionizing radiation and ultraviolet radiation

All cells were seeded 18–24 hours prior to radiation. For IR, cells were irradiated with 6 Gray (6 Gy) using a Gammacell Co60 self-shield irradiator (Cross Cancer Institute, Department of Experimental Oncology, University of Alberta). Cells were seeded 18–24 hours prior to radiation. Before UVC exposure, cells were washed with PBS. Cells were exposed to ultraviolet-visible (UVC) radiation using a Spectroline ® UV crosslinker (Fisher Scientific, USA). After exposure, PBS was discarded, and new media was added. The cells were then returned to the incubator.

### Total protein extraction, SDS-PAGE, and immunoblotting

To lyse cells, an appropriate amount of 1% NP 40 lysis buffer supplemented with 1X protease inhibitor cocktail (Roche) was used (50 mM Tris-HCl, pH 8.0, 150 mM NaCl, 1 mM EDTA, pH 8.0, and 1% NP-40). Cells were then sonicated and centrifuged at high speed at 4 °C for 15 minutes. The clear cell lysates that contained the total proteins were then moved to new tubes. The Bio-Rad protein assay was performed to quantify protein concentrations (Bio-Rad Laboratory Inc., USA). Proteins were mixed with equal volumes of 2X loading buffer (0.1 Tris-HCl pH 6.8, 20% glycerol, 4% SDS, and 0.2% bromophenol blue). The proteins were then denatured by incubation at 100 °C for 5 minutes. SDS-polyacrylamide gel electrophoresis was conducted to separate the proteins from the total protein lysates. The separated proteins in the gels were then transferred to polyvinylidene difluoride (PVDF) membranes (Immobilon® -P transfer membranes, EMD Millipore Corp., USA).

The PVDF membrane was incubated with a blocking solution (5% milk dissolved in TBST) on a shaker for 1 hour at room temperature (RT). After the blocking step, the membrane was washed 3 times for 10 minutes with TBST (200 mM Tris, 150 mM NaCl dissolved in ddH2O, pH 7.4, plus 1% Tween 20) and incubated with primary antibody (diluted in 1X TBS) either for 1.5 hours at RT or overnight at 4 °C on a shaker. The membrane was washed with ddH2O for a few seconds and then 3 times for 5 minutes each with TBST. The membrane was then incubated with the complementary secondary antibody (diluted in 1% milk-TBST) at RT for one hour. Finally, the membrane was washed three times for 10 minutes each with TBST. In the dark, the membrane was treated with an ECL solution for 1 minute (Western Lightning TM Plus-ECL-GE Healthcare, UK). An X-ray film (Fuji Film Corp., Japan) was placed on the membrane for different exposure durations. Then, the signal was developed using the Optimax X-ray Film Producer (Protec, USA).

### Primary antibodies

All primary and secondary antibodies used to detect their targets are listed in Table 1.

**Table 1:** List of all antibodies.

Antibody	Dilution	Source	secondary
P53 (Pab1801)	1:100	Santa Cruz Biotechnology	Mouse
P53 (Pab421)	1:100	Santa Cruz Biotechnology	Mouse
P53 (DO-1)	1:1000	Santa Cruz Biotechnology	Mouse
UBE4B	1:500	BD Biosciences/Santa Cruz Biotechnology	Mouse
HDM2 (Smp14)	1:100	BD Biosciences	Mouse
HDM2 (2A10)	1:100	EMD Biosciences	mouse
Wip1	1:500	Santa Cruz Biotechnology, INC.	Rabbit
ATM	1:1000	Cell Signaling Technology	Rabbit
ATR	1:1000	Cell Signaling Technology	Rabbit
Flag (M5)	1:1000	MilliporeSigma	Mouse
Anti-HA	1:20	12CA5; Roche	Mouse
Phos-UBE4B S669	1:200	GeneScript USA INC.	Rabbit
Actin	1:3000-5000	MilliporeSigma	mouse

### Transient and stable transfection

All transfections were performed using calcium phosphate-based methods. The constructs included Flag-Wip1 (cloned in p3XFlag-CMV.10, Sigma, USA), HA-tagged ubiquitin, pCMV-BAM-HDM2, Flag-tagged UBE4B, Flag-tagged C500, Flag-tagged C800, pcDNA3.1-p53, shRNA-UBE4B-1 (position 3063 of human UBE4B cloned in pSUPER.gfp.neo). Cells were harvested 40 hours after transfection. Cells were trypsinized and treated with geneticin (G418) (Thermo Fisher Scientific) to generate stable clones. The colonies were allowed to grow for two weeks. For the viral transduction vector, the pSUPER.retro.puro vector (Oligoengine, USA) was used. Oligos (5-GAT CCC CGG ATG ACT TTG TCA GAG CTT TCA AGA GAA GCT CTG ACA AAG TCA TCC TTT TTA-3) were cloned into the vector following the manufacturer’s instructions.

### Retroviral infection/transmission

To generate retroviral supernatants, the Wip1-pSUPER.retro.pure plasmid was transfected using the calcium phosphate method in a packaging cell line (HEK-239T). The viral supernatants were collected 48 hours after transfection. Retroviral supernatants were filtered and stored in a freezer at −80 °C. Wip1 retroviral supernatants and 4–8 μg/ml polybrene were added to cells for 6-18 hours to infect the cells. Cells were then washed with PBS, replenished with fresh media, and allowed to recover for 24 hours. To establish stable clones, infected cells were treated with 1–2 μg/ml puromycin and selected for two weeks.

### Coimmunoprecipitation analysis

Briefly, total protein extraction was performed as previously described. Approximately 600–1000 micrograms of total protein were mixed with a protease inhibitor cocktail and an appropriate amount of antibody. The mixture was then incubated on ice for one hour. The protein-antibody mix was added to protein A/G PLUS-agarose beads (Santa Cruz, USA). The mixture was incubated at 4 °C on a rocker overnight. The mixture was spun at 2500 rpm for 2 minutes, and the supernatant was discarded. The pellet beads were washed several times. The loading buffer was added to the beads and then incubated at 100 ° C for six minutes. The tube was then spun at high speed. The supernatants containing the denatured proteins were separated by SDS-PAGE and transferred to PVDF. Approximately 30–50 μg of total protein lysates were used as input (direct immunoblotting of total protein from each sample).

### In vivo ubiquitination analysis

An in vivo ubiquitination assay was performed to determine a target protein’s amount and level of ubiquitination. Cells were transfected with specific constructs plus HA-tagged ubiquitin. Thirty-six hours after transfection, cells were harvested and immunoprecipitated with the indicated antibodies. Immune complexes recovered with protein A-Sepharose were washed four times with radioimmunoprecipitation assay buffer [RIPA buffer: 50 mM Tris HCl (pH7.6), 150 mM NaCl, 1.0% (v/v) NP-40, 0.5% (w/v) Sodium Deoxycholate, 1.0 mM EDTA, 0.5 mM EGTA, 1.0% (w/v) SDS, and protease inhibitor tablet (Roche)], separated on 10% SDS-PAGE, and analyzed by immunoblotting as described previously [70].

### RNA extraction, RT–PCR, and quantitative real-time PCR

The method for RNA extraction and analysis was previously described [71]. Briefly, cells were exposed to UV light and harvested at the indicated time points after treatment. Total RNA was extracted using TRIzol (Invitrogen). The first-strand cDNA for real-time PCR was synthesized from 2 µg of total RNA using SuperScript (Invitrogen). Real-time PCR was conducted with the StepOnePlus Real-Time PCR System utilizing the SYBR Green PCR master mix (Applied Biosystems). The primers used for quantitative PCR (qPCR) were as follows: human Wip1 forward: 5’-TGGGTGAGCATGGACAATCT-3’; human Wip1 reverse: 5’-GGTGGTGTAGAACATGGGA-3’; human GAPDH forward: 5’-AGCCACATCGCTCAGACAC-3’; and human GAPDH reverse: 5’-GCCCAATACGACCAAATCC-3’. The mRNA expression levels of Wip1 were normalized to GAPDH, and relative expression was calculated compared to untreated samples using the comparative Ct method, as previously described [71].

### Colony formation assay

A colony formation assay was performed to determine the effect of inhibition or overexpression of targeted proteins on cell proliferation and viability. Thirty-six hours after transfection with the targeted plasmids (for transiently transfected cells), and then 500 cells were seeded in plates (60 mm). For stable clones, cells were seeded, transfected with indicated expressing plasmids and selected with G418 for two weeks. Colonies were fixed with 100% methanol and stained with 0.5% crystal violet. The colonies were observed and counted under an inverted microscope. Data are presented as images of the plates and graphs of the ratio of colony formation. This ratio was calculated as follows: the number of colonies divided by the number of seeded cells.

### Microscopy

Plates were observed under an EVOS M5000 imaging system to visualize and image-treated cultured cells (Thermo Fisher Scientific, USA).

### Statistical analysis

The two-tailed Student’s *t*-test was used to analyze the statistical significance of the data. Statistical significance was expressed as a *p*-value, where a *p*-value less than 0.05 was considered significant using the GraphPad Prism 8 software.

## Supplementary information

full length uncropped original western blots

## Data Availability

All other relevant data supporting the findings of this study are available in the article.
