# Supplementary material for: Deciphering UBE4B phosphorylation dynamics: a key mechanism in p53 accumulation and cancer cell response to DNA damage
Source: Cell Death Discov. 2025 Apr 2;11:131. doi: 10.1038/s41420-025-02441-9 (PMC11965332; doi:10.1038/s41420-025-02441-9)

Figure 1A

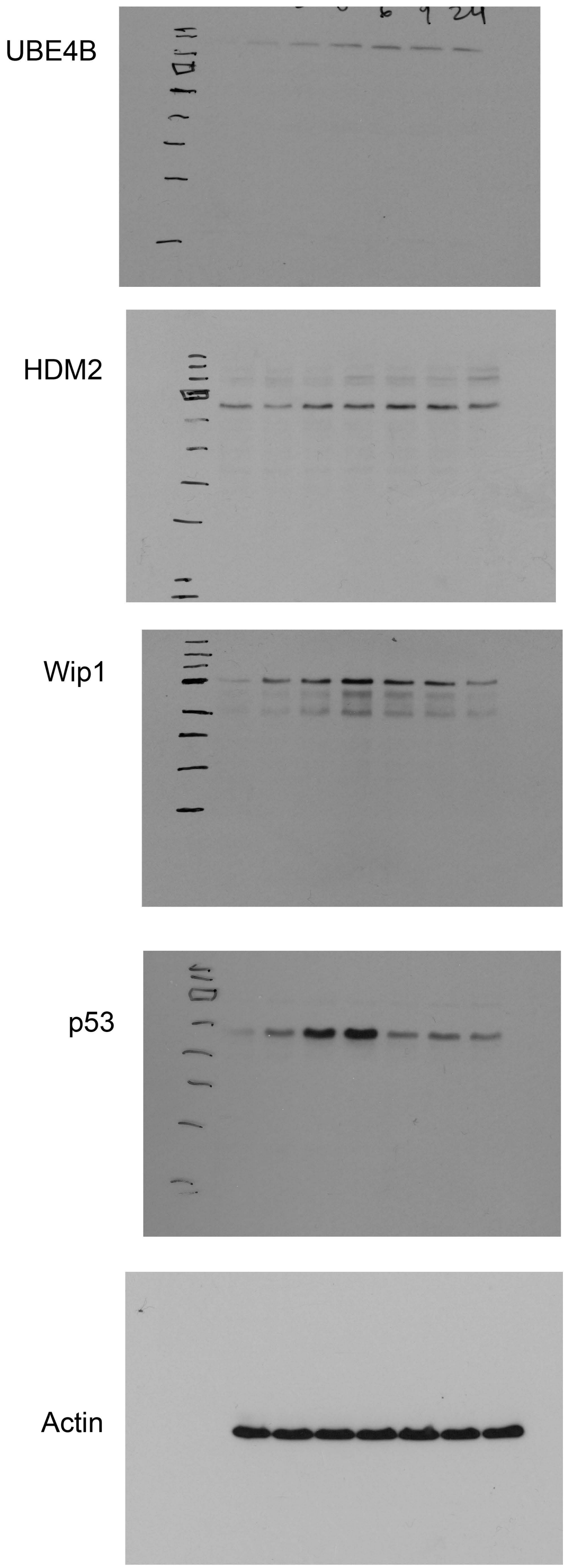

Figure 1B

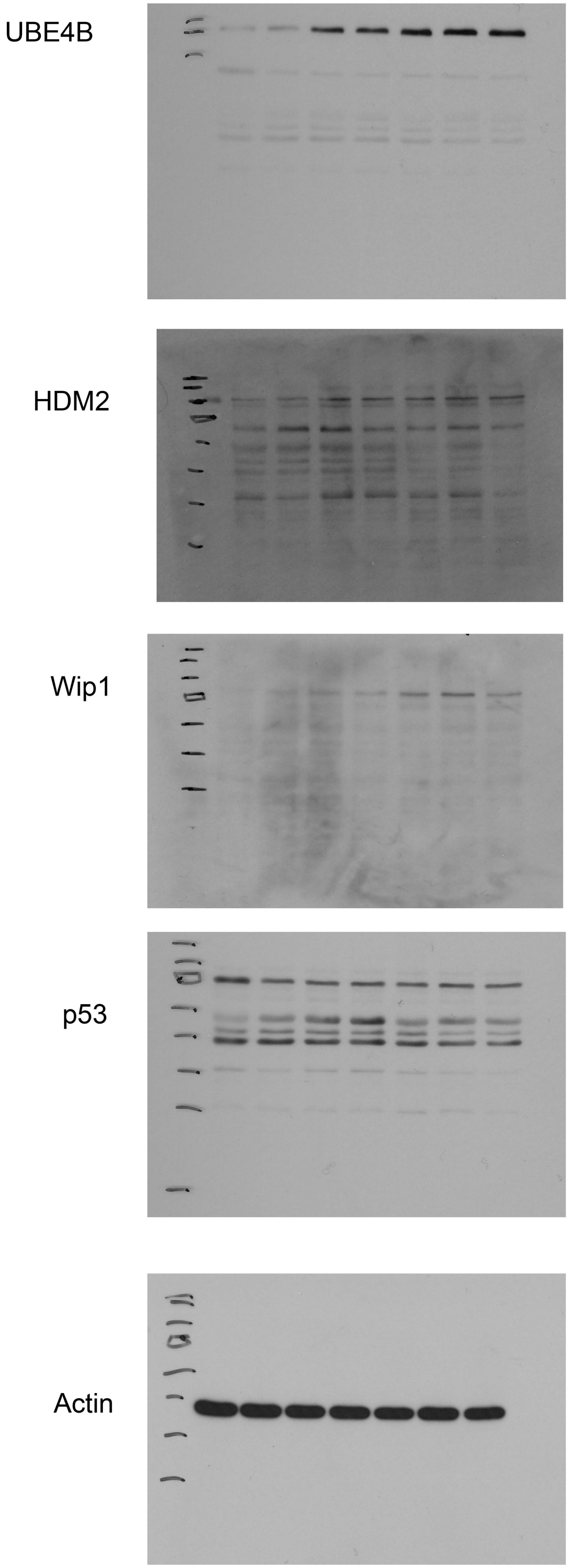

Figure 1C

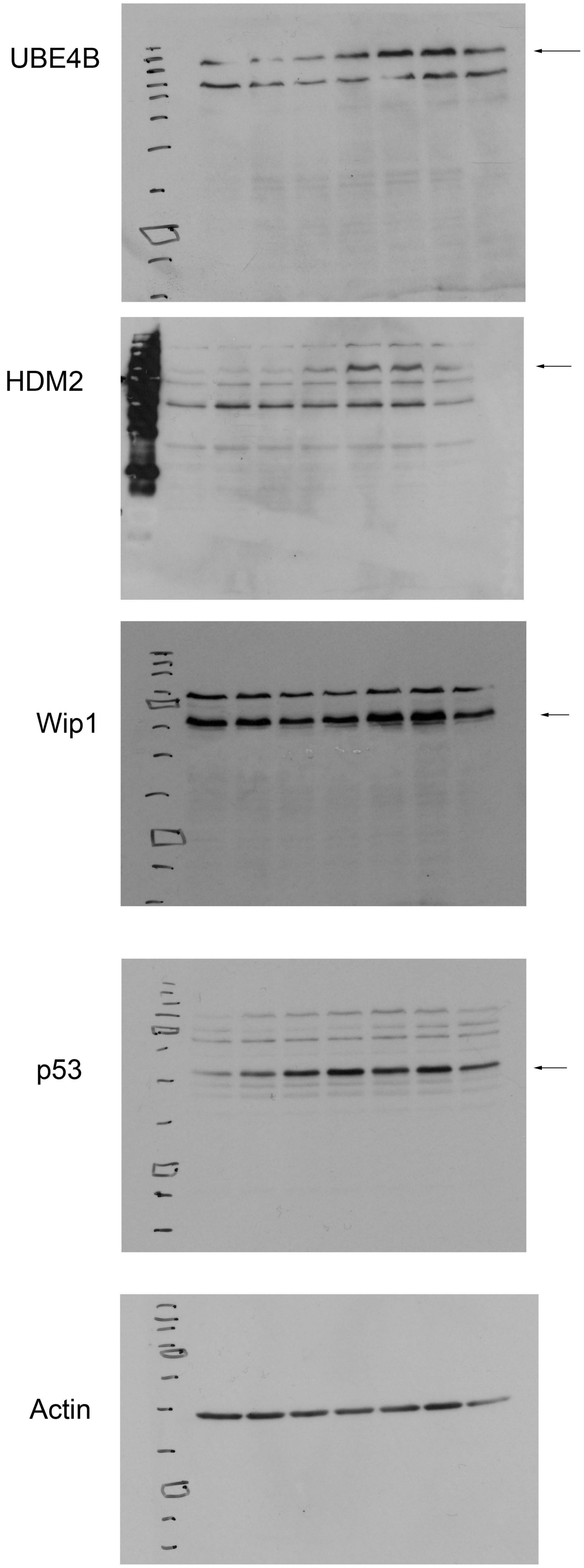

Figure 1D

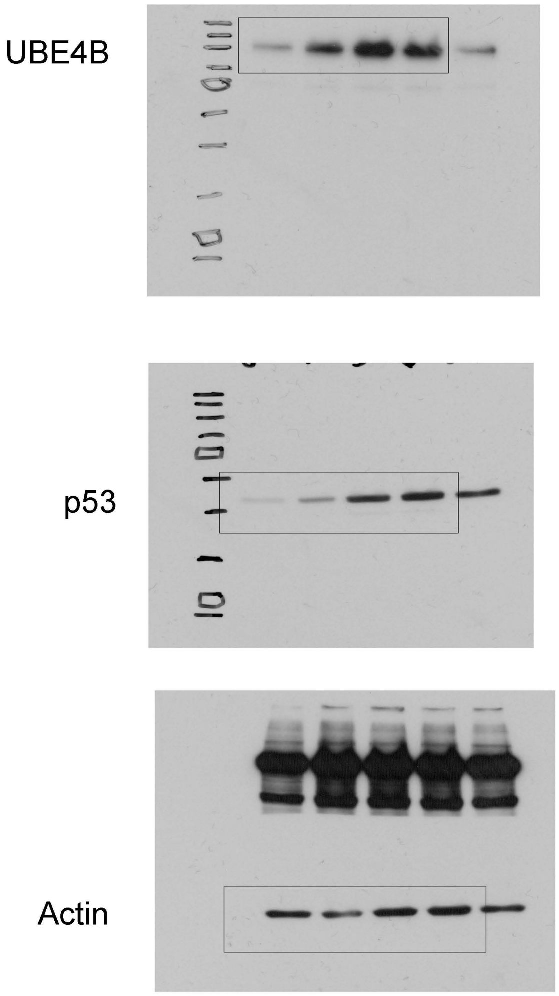

Figure 1E

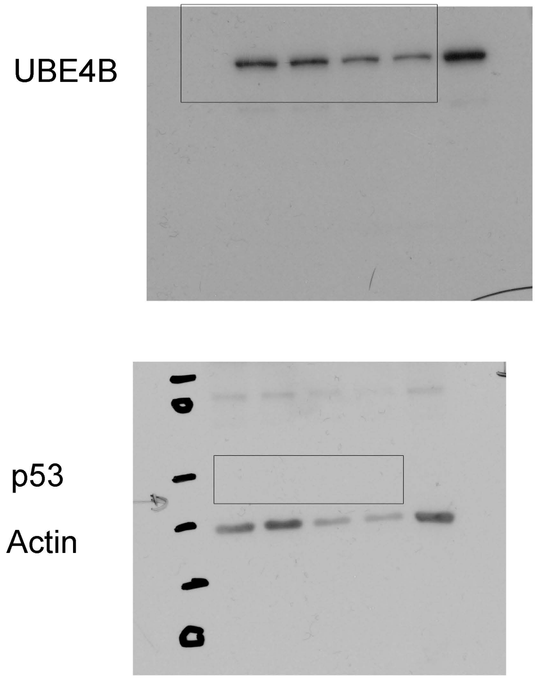

Figure 1F

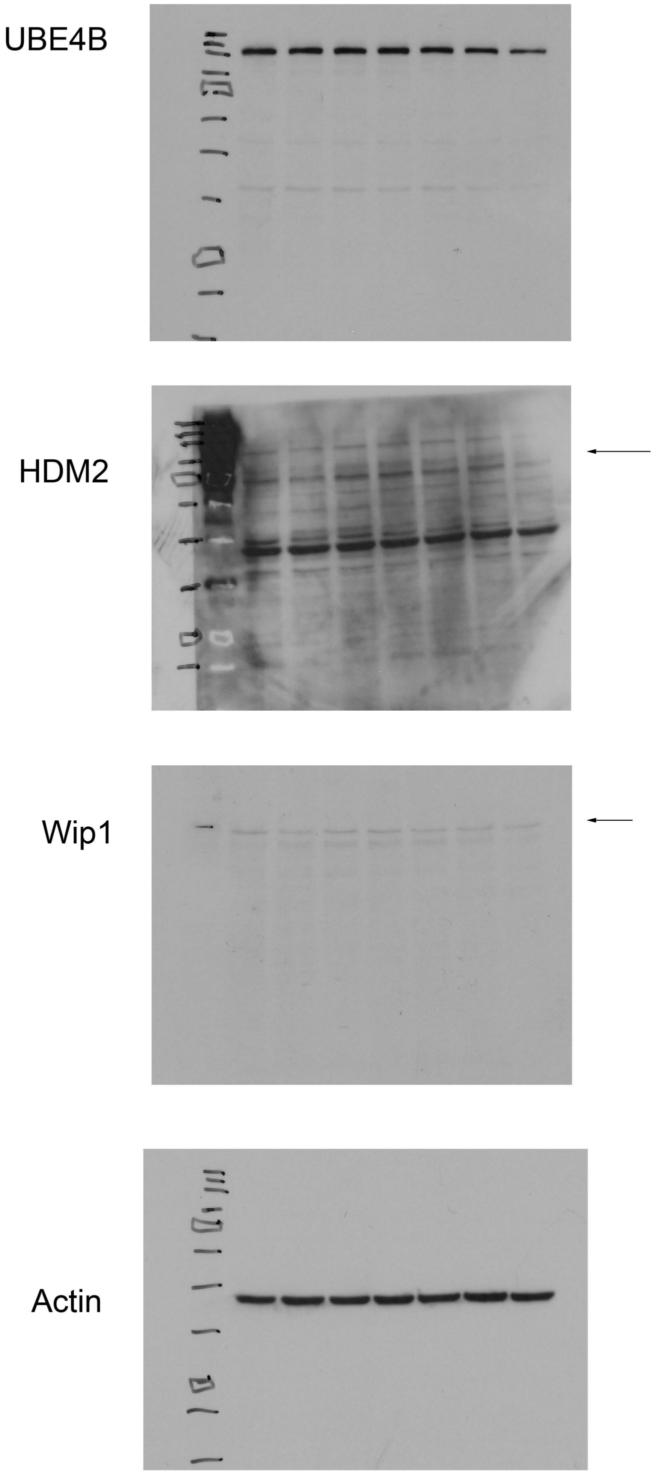

Figure 1G

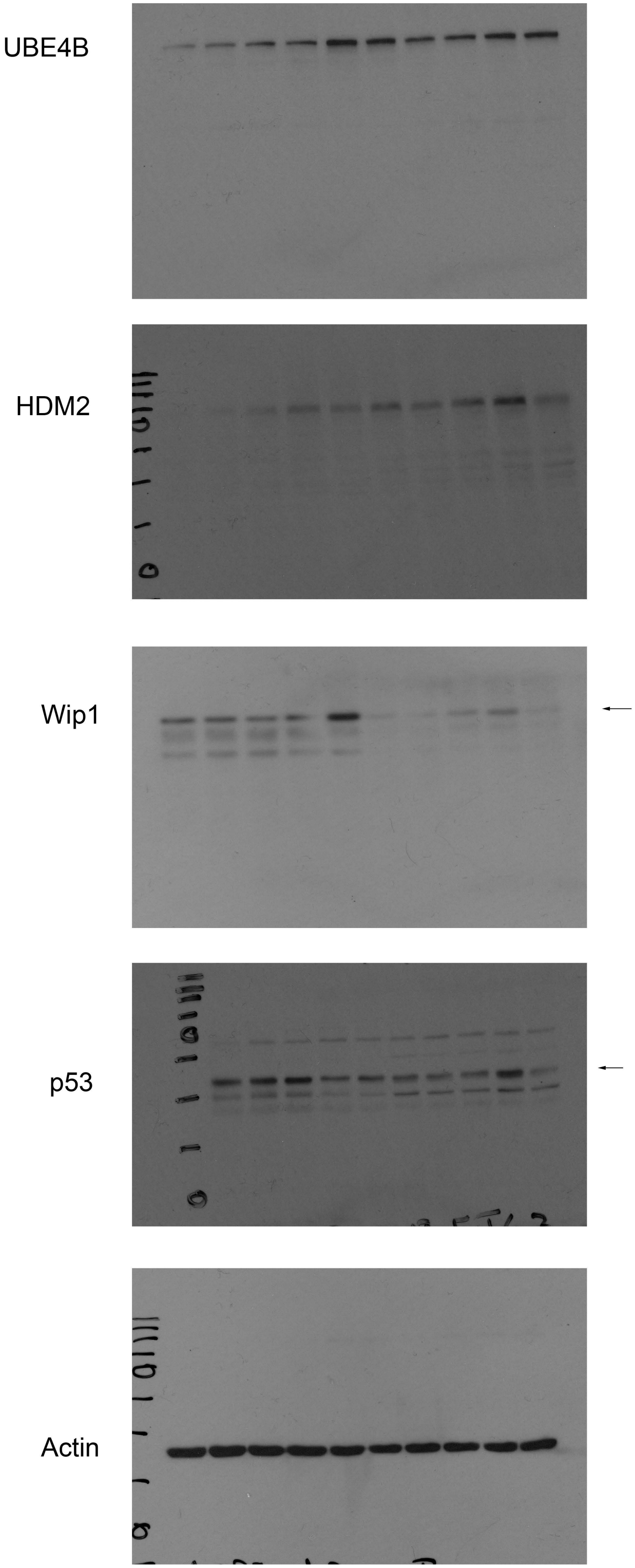

Figure 2A

UBE4B

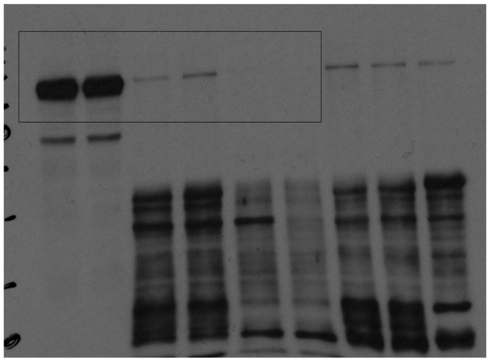

p53

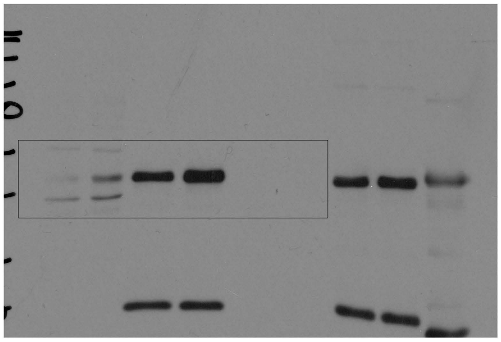

actin

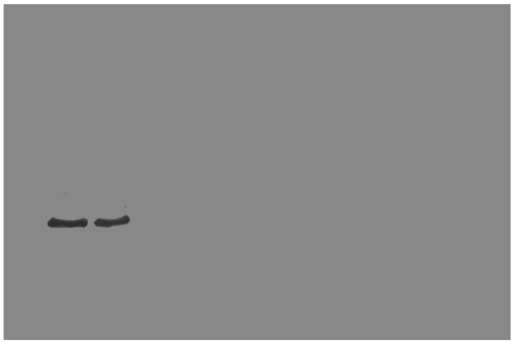

Figure 2B

Wip1

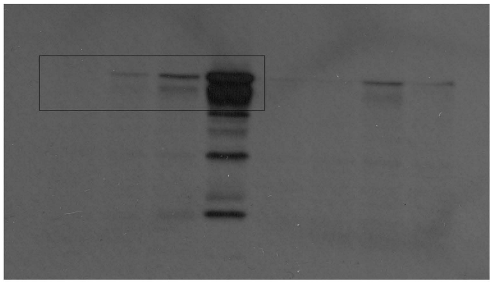

Figure 2B

UBE4B

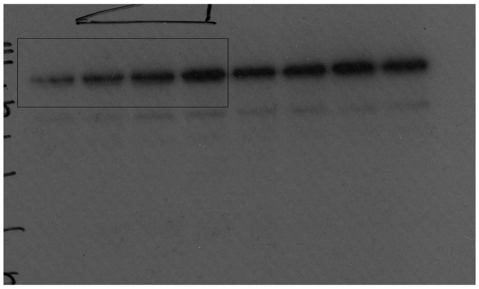

Actin

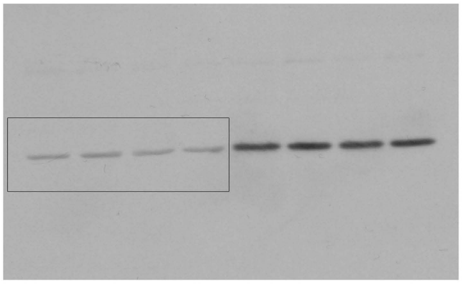

Figure 2C

Wip1

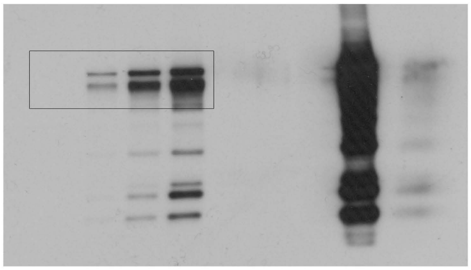

UBE4B

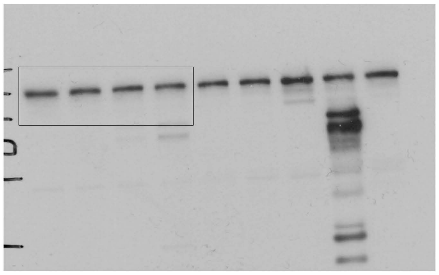

Actin

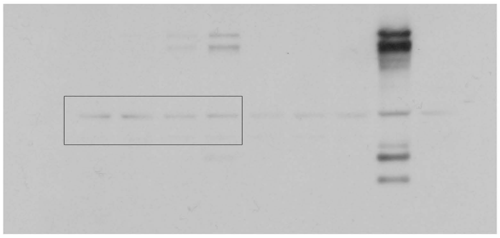

Figure 2D

Wip1

UBE4B

HDM2

p53S15

p53

actin

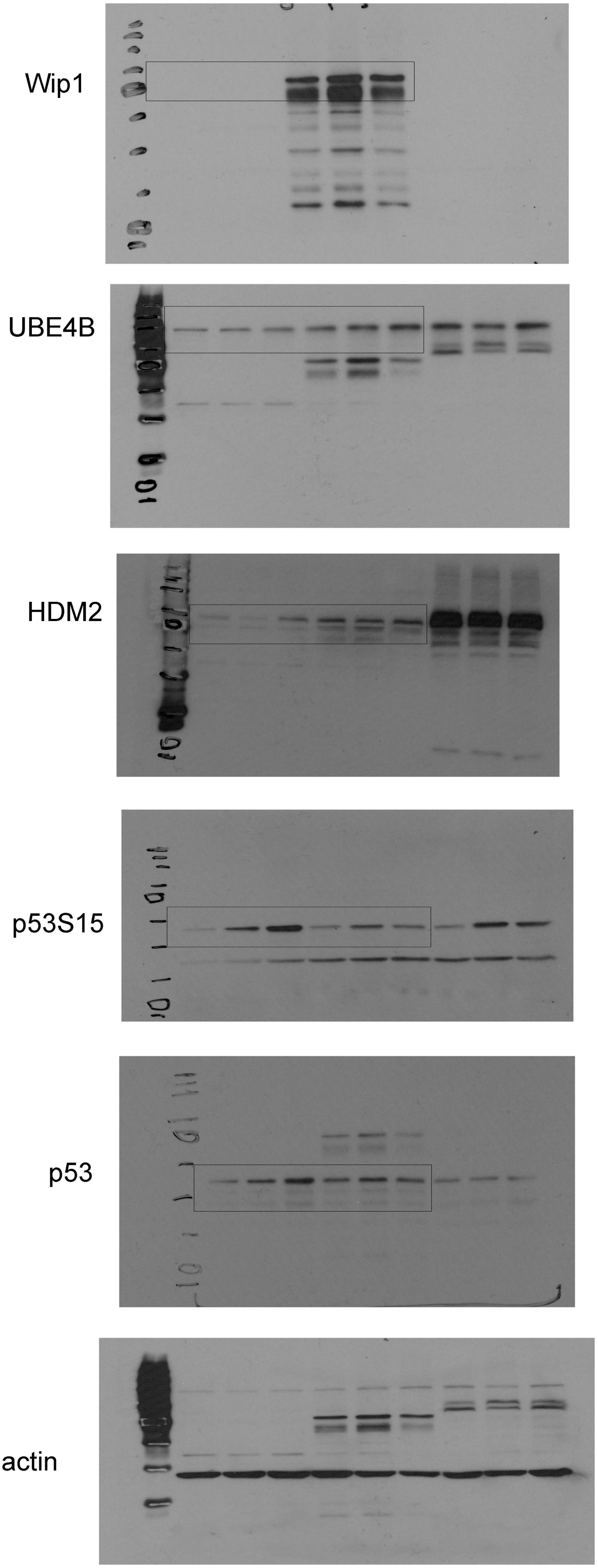

Figure 3A

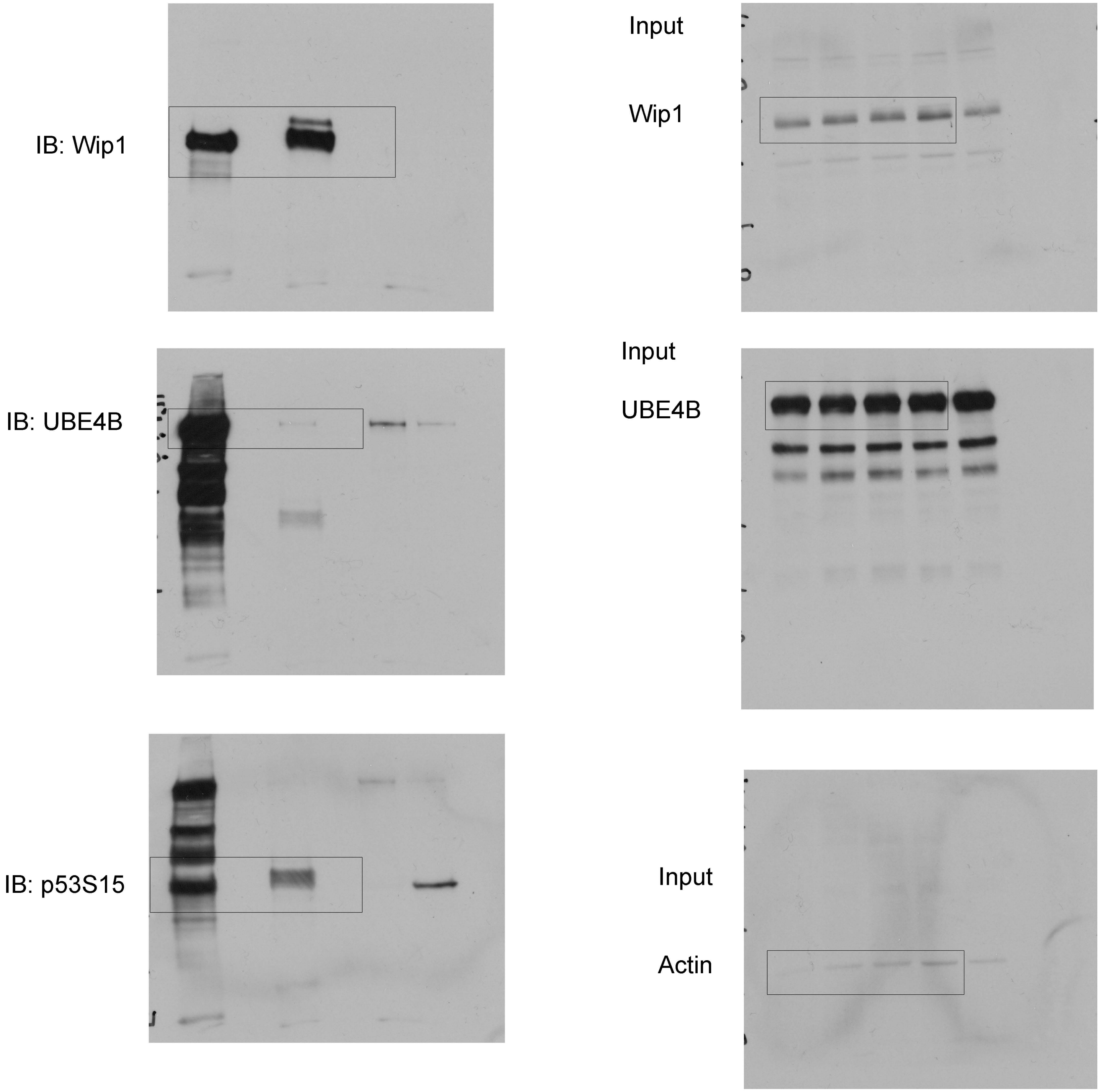
IB: Wip1
Input
Wip1
IB: UBE4B
Input
UBE4B
IB: p53S15
Input
Actin

Figure 3C

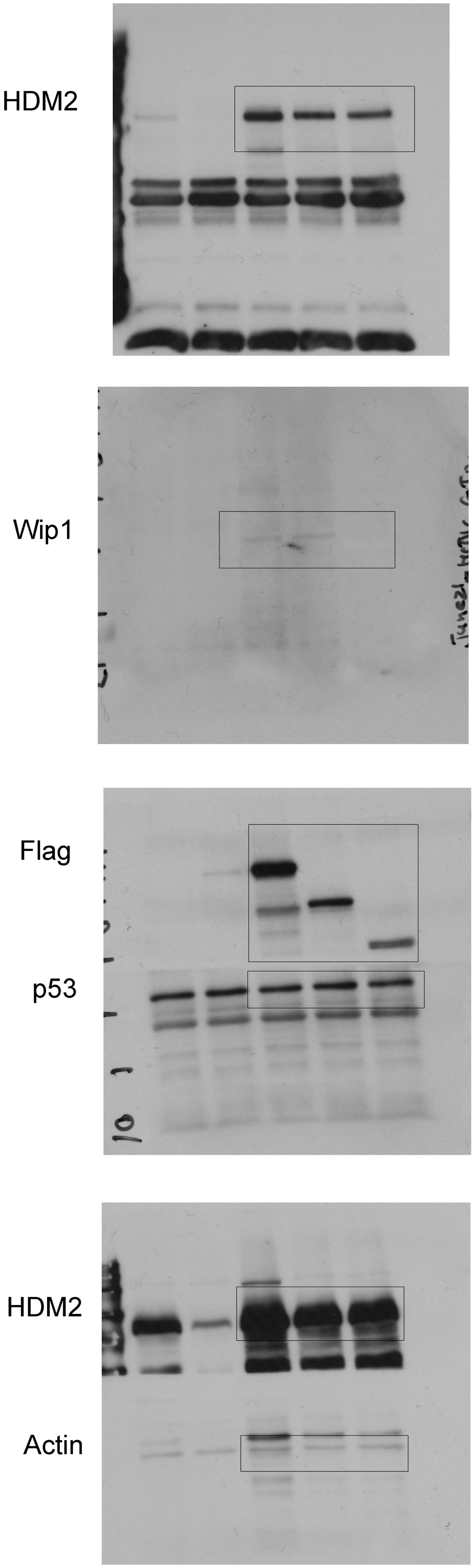

Figure 3D

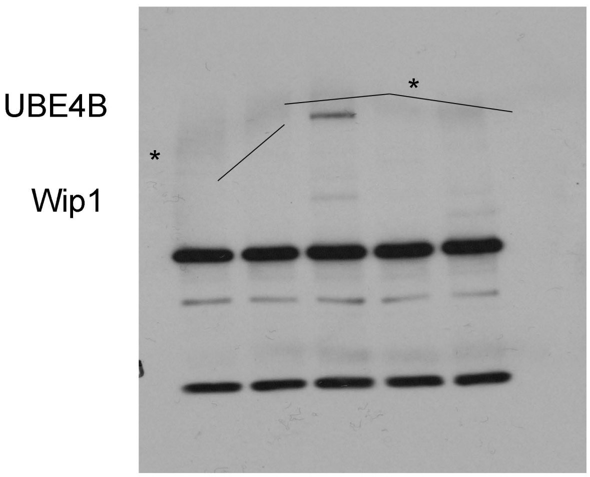

Figure 3G

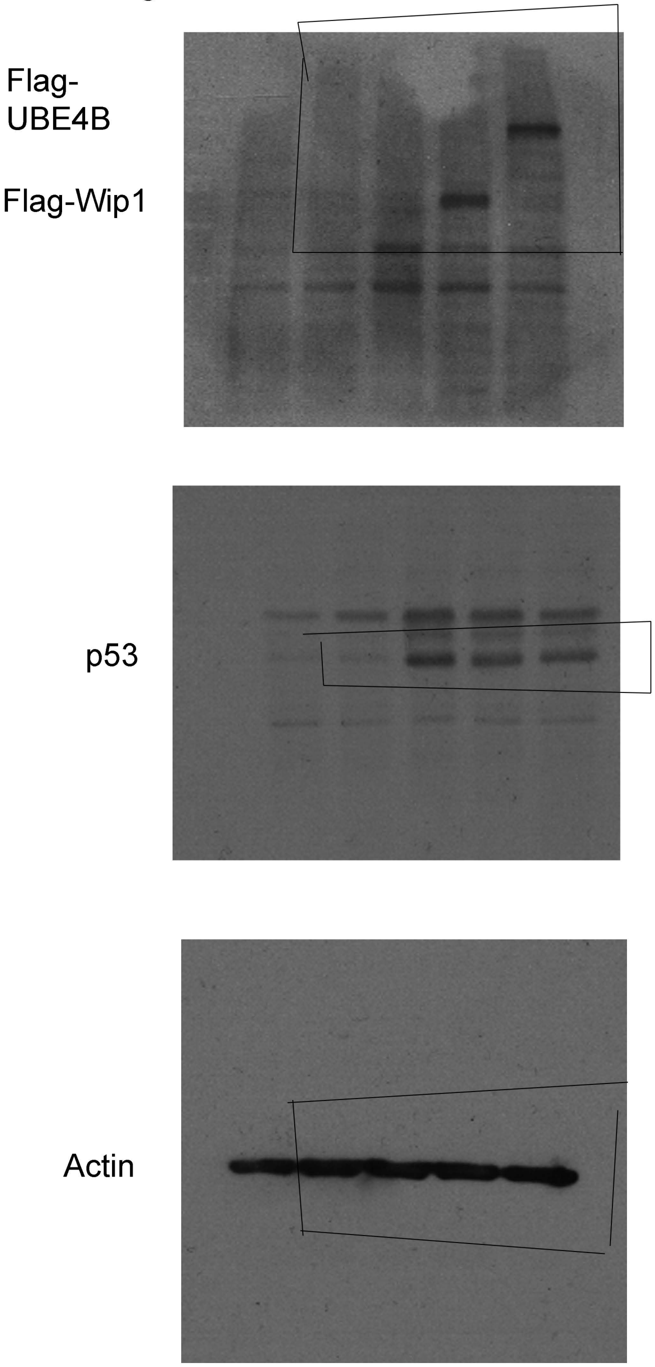

Figure 4A

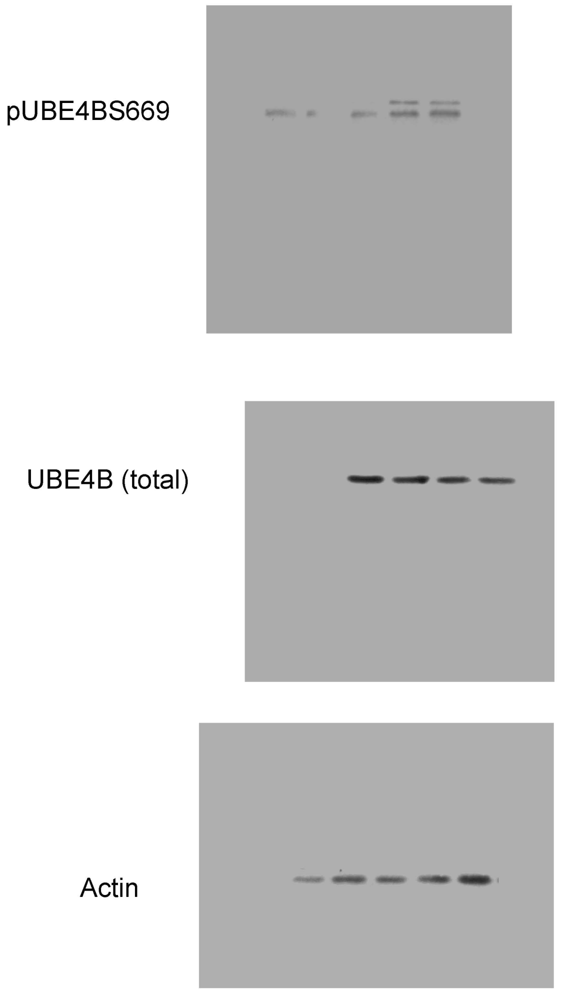

Figure 4B

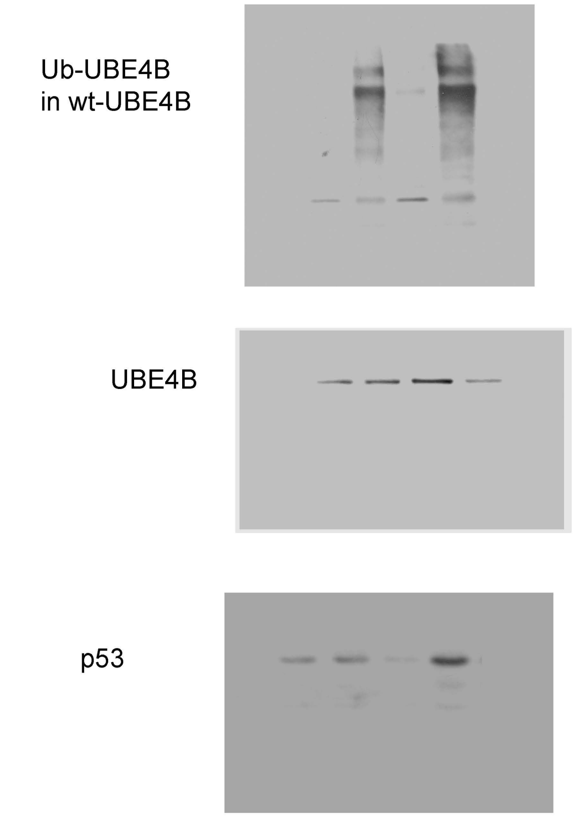

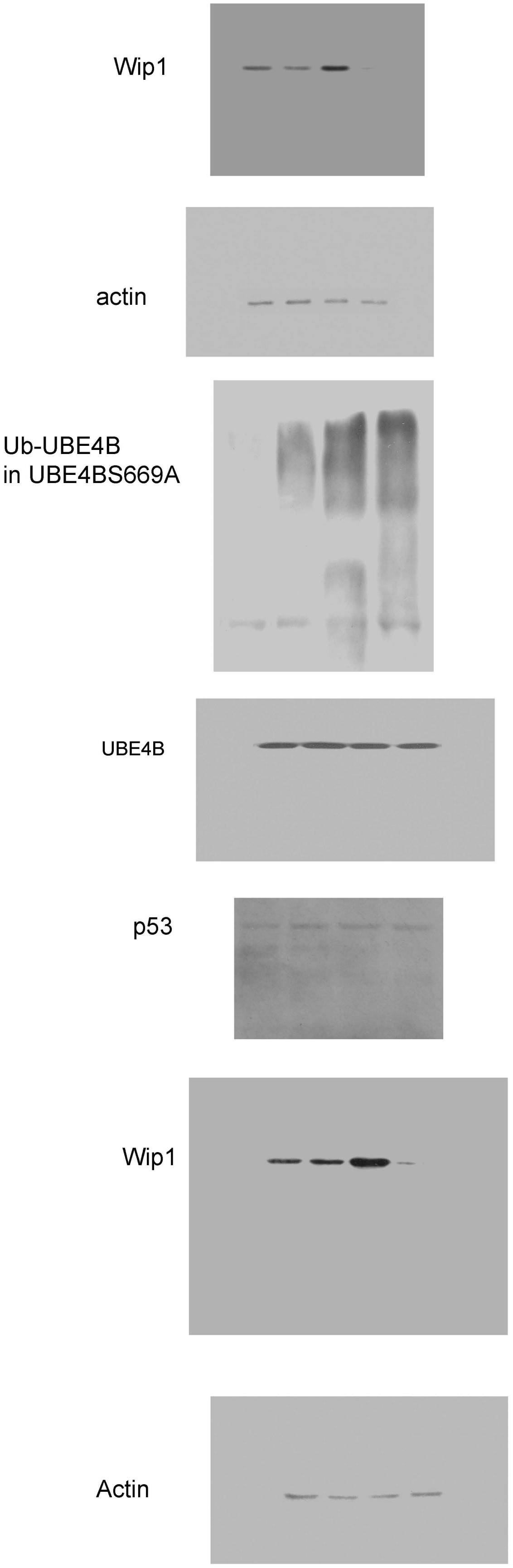

Fig-4C

UBE4B

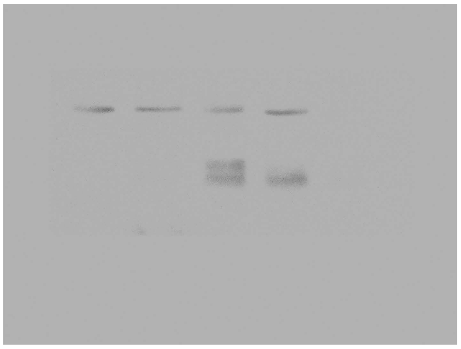

Actin

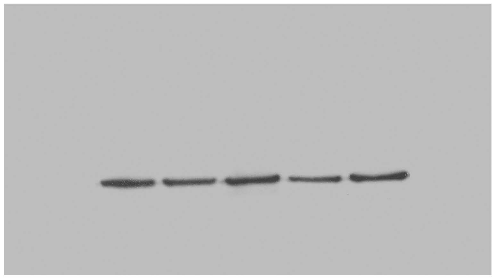

UBE4B

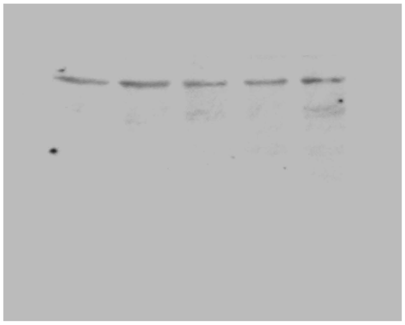

Actin

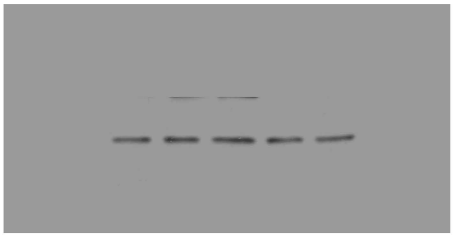

Fig-4D

UBE4B

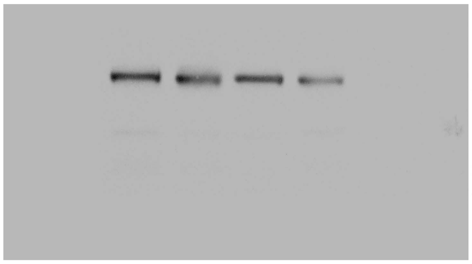

Actin

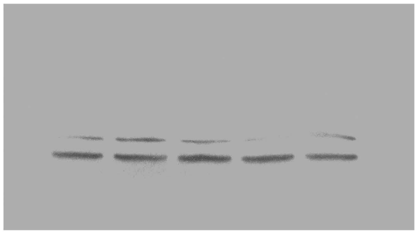

UBE4B

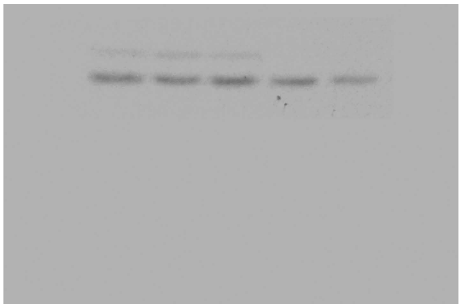

Actin

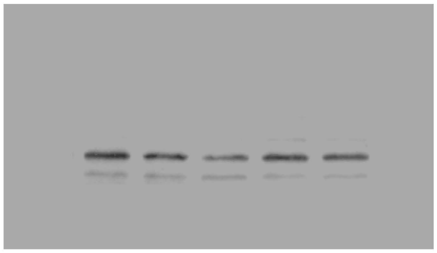

Fig-4E

UBE4B

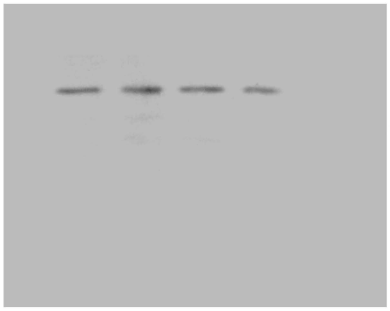

Actin

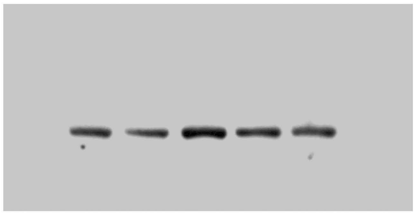

UBE4B

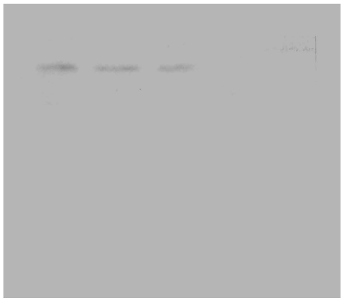

Actin

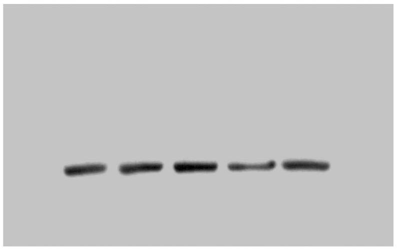

Figure 5A

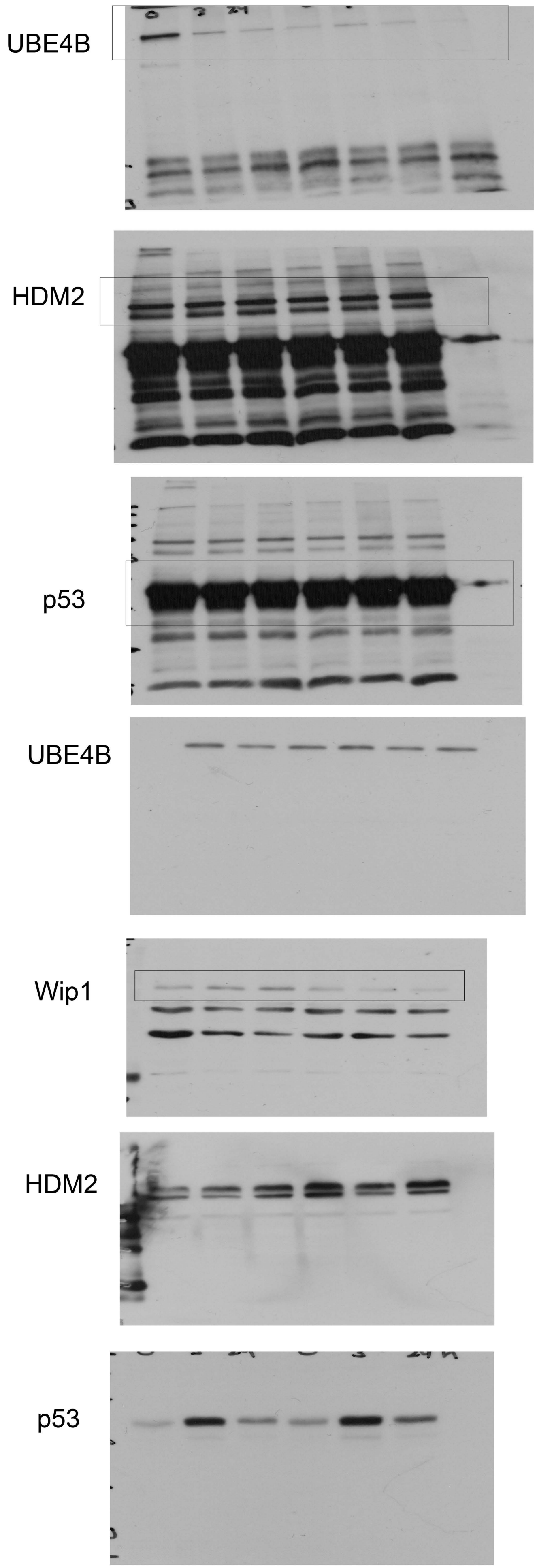

Figure 5B

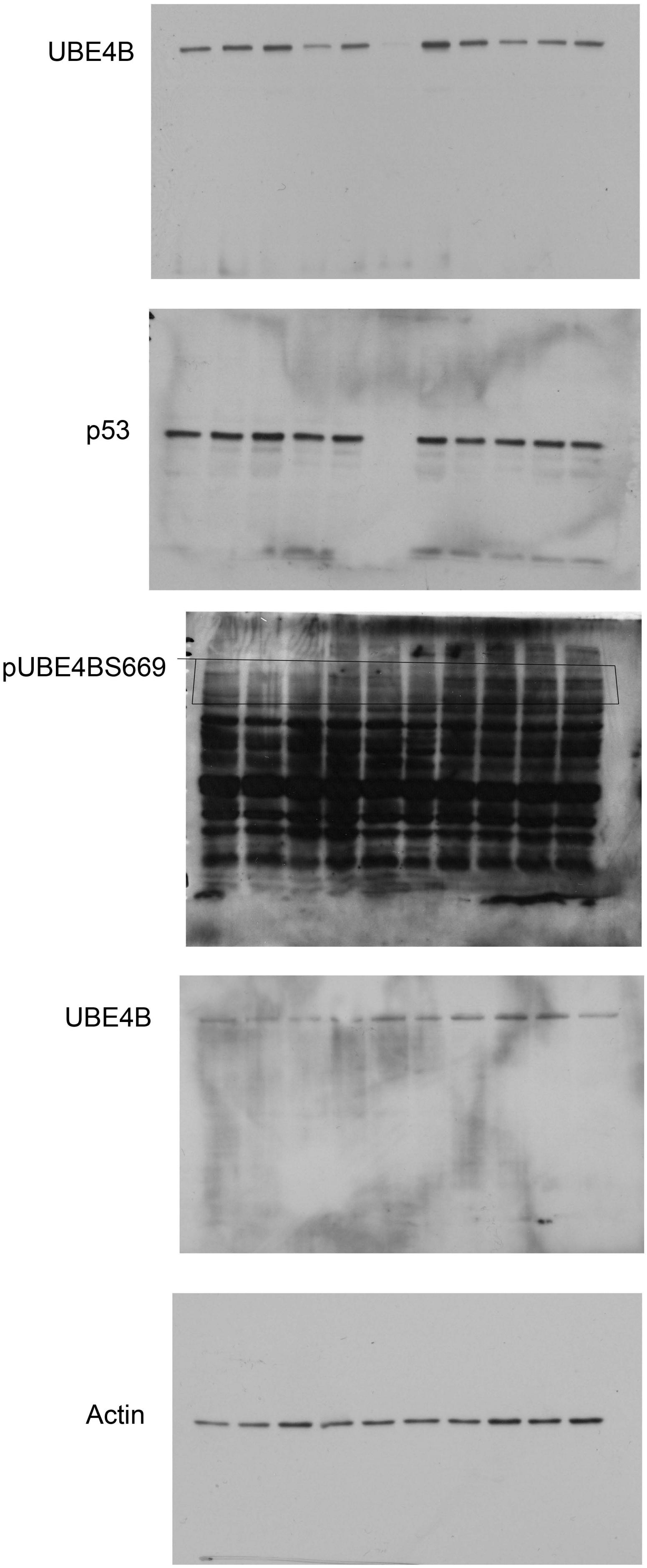

Figure 5C

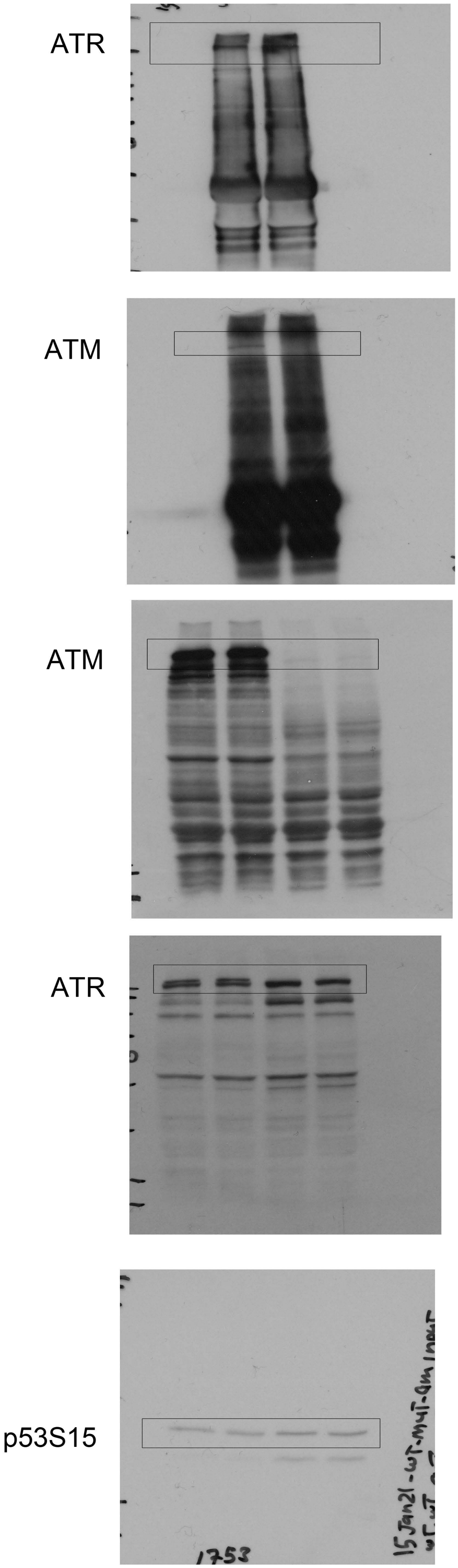

Figure 5D

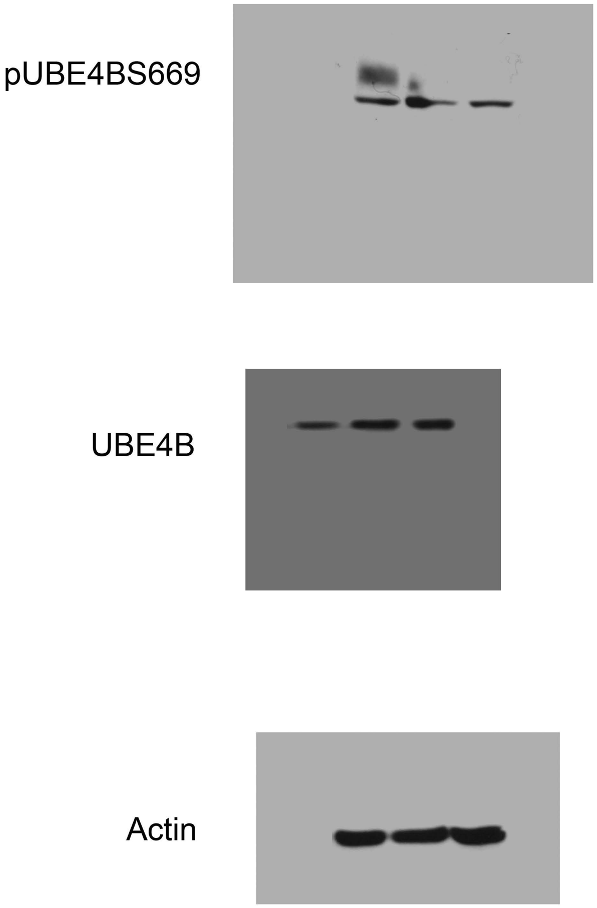

Figure 5E

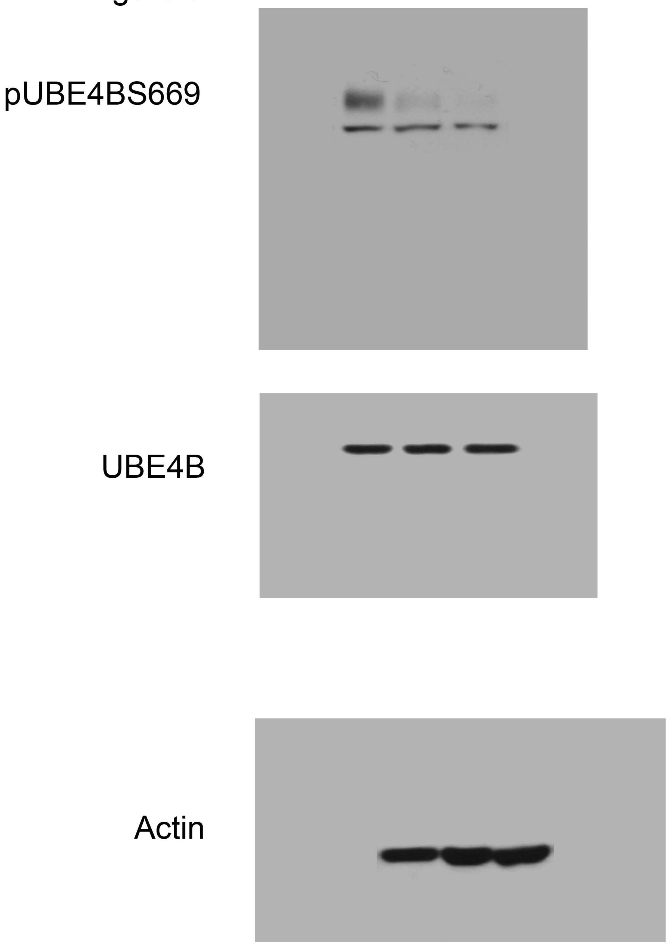

Figure 5F

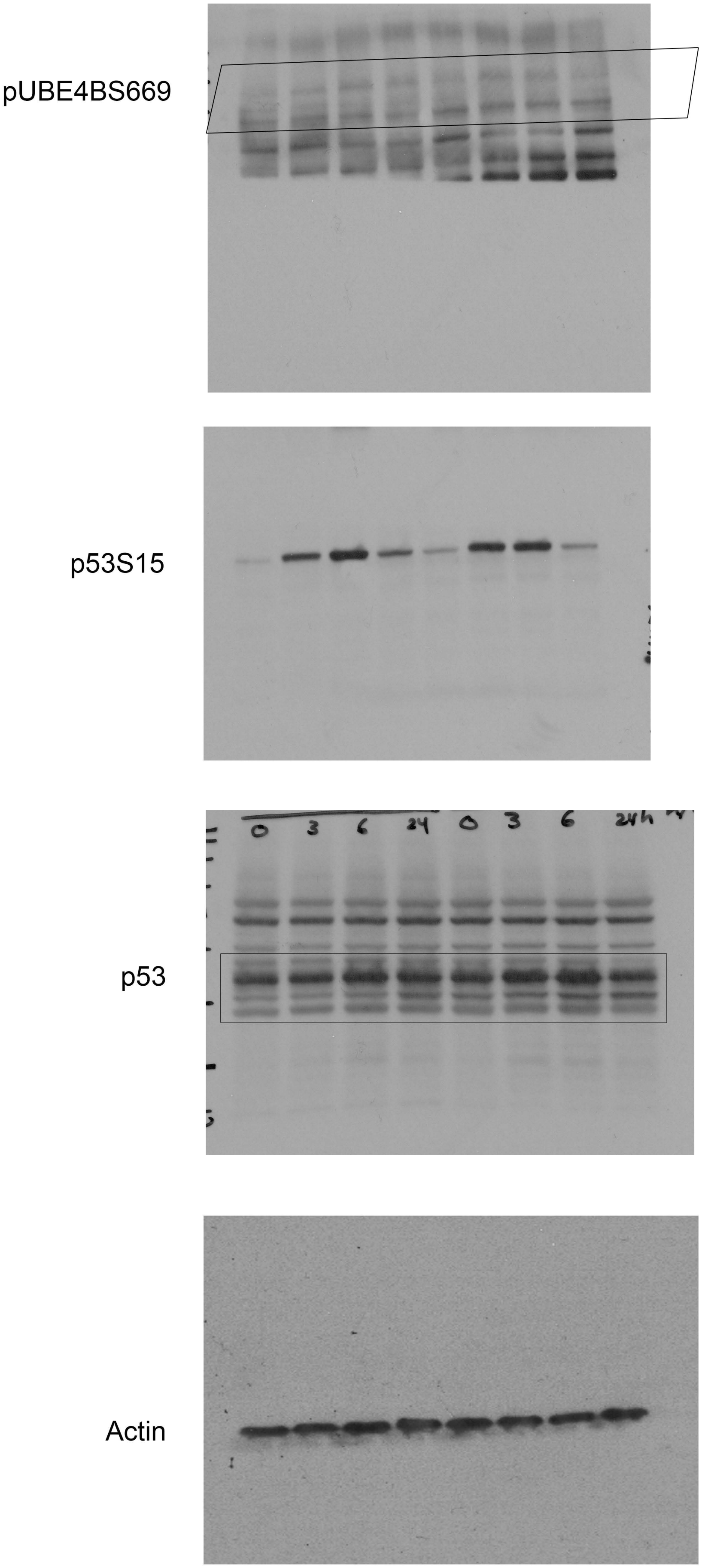

Figure 6A

UBE4B

Actin

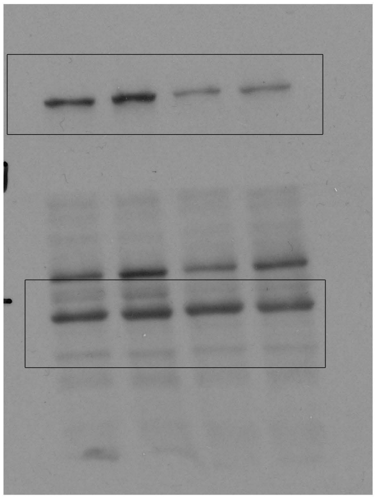

Wip1

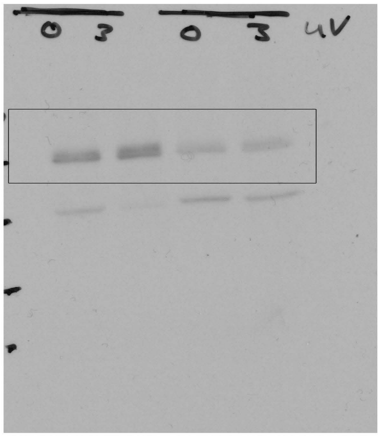

Figure 6B

pUBE4BS669

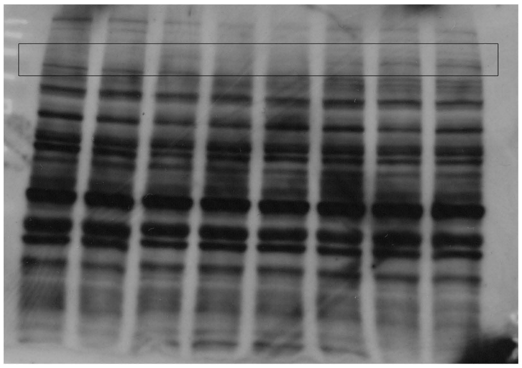

UBE4B

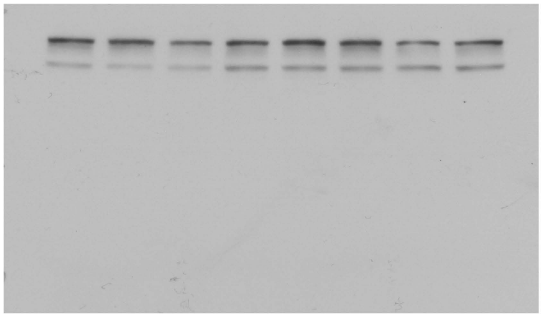

Wip1

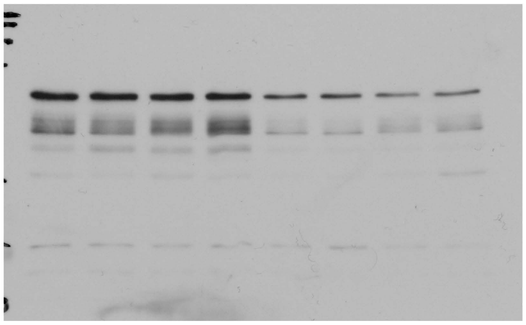

p53S15

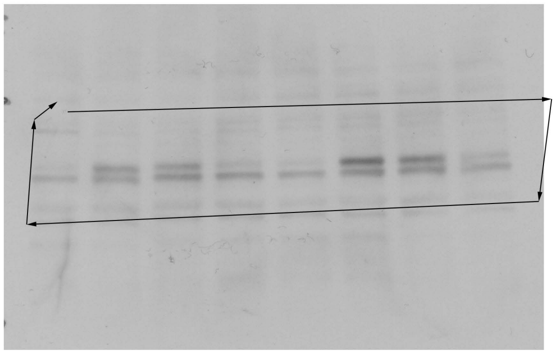

p53

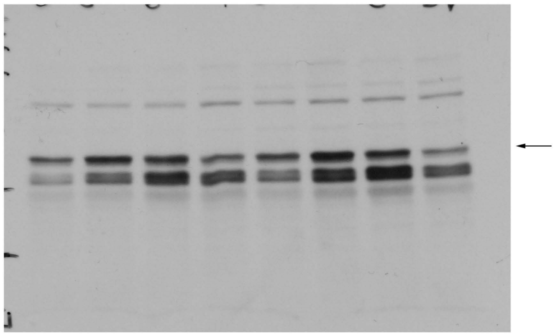

Actin

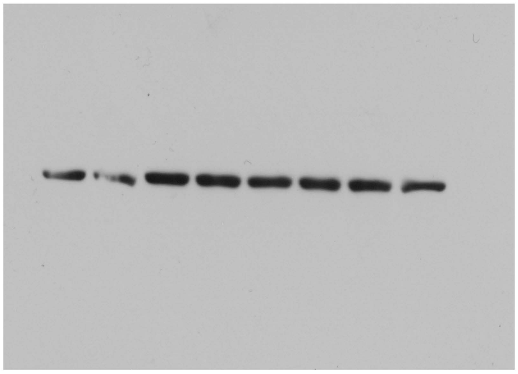

Supplement: Supplementary file 1 — full length uncropped original western blots [file 41420_2025_2441_MOESM1_ESM.pdf]
